# Near‐Infrared Organic Photodetectors toward Skin‐Integrated Photoplethysmography‐Electrocardiography Multimodal Sensing System

**DOI:** 10.1002/advs.202304174

**Published:** 2023-11-22

**Authors:** Zirui Lou, Jun Tao, Binbin Wei, Xinyu Jiang, Simin Cheng, Zehao Wang, Chao Qin, Rong Liang, Haotian Guo, Liping Zhu, Peter Müller‐Buschbaum, Hui‐Ming Cheng, Xiaomin Xu

**Affiliations:** ^1^ Shenzhen International Graduate School & Tsinghua‐Berkeley Shenzhen Institute Tsinghua University Shenzhen 518055 China; ^2^ School of Advanced Materials Peking University Shenzhen Graduate School Shenzhen 518055 China; ^3^ Lehrstuhl für Funktionelle Materialien Physik Department Technische Universität München James‐Franck‐Str. 1 85748 Garching Germany; ^4^ State Key Laboratory of Silicon and Advanced Semiconductor Materials School of Materials Science and Engineering Zhejiang University Hangzhou 310027 China; ^5^ Heinz Maier‐Leibnitz‐Zentrum (MLZ) Technische Universität München Lichtenbergstr. 1 85748 Garching Germany; ^6^ Institute of Technology for Carbon Neutrality & Faculty of Materials Science and Energy Engineering Shenzhen Institute of Advanced Technology Chinese Academy of Sciences Shenzhen 518055 China; ^7^ Shenyang National Laboratory for Materials Science Institute of Metal Research Chinese Academy of Sciences Shenyang 110016 China

**Keywords:** flexible (opto)electronics, multimodal biosensing, near‐infrared organic photodetectors, skin‐integrated electronics

## Abstract

In the fast‐evolving landscape of decentralized and personalized healthcare, the need for multimodal biosensing systems that integrate seamlessly with the human body is growing rapidly. This presents a significant challenge in devising ultraflexible configurations that can accommodate multiple sensors and designing high‐performance sensing components that remain stable over long periods. To overcome these challenges, ultraflexible organic photodetectors (OPDs) that exhibit exceptional performance under near‐infrared illumination while maintaining long‐term stability are developed. These ultraflexible OPDs demonstrate a photoresponsivity of 0.53 A W^−1^ under 940 nm, shot‐noise‐limited specific detectivity of 3.4 × 10^13^ Jones, and cut‐off response frequency beyond 1 MHz at −3 dB. As a result, the flexible photoplethysmography sensor boasts a high signal‐to‐noise ratio and stable peak‐to‐peak amplitude under hypoxic and hypoperfusion conditions, outperforming commercial finger pulse oximeters. This ensures precise extraction of blood oxygen saturation in dynamic working conditions. Ultraflexible OPDs are further integrated with conductive polymer electrodes on an ultrathin hydrogel substrate, allowing for direct interface with soft and dynamic skin. This skin‐integrated sensing platform provides accurate measurement of photoelectric and biopotential signals in a time‐synchronized manner, reproducing the functionality of conventional technologies without their inherent limitations.

## Graphical abstract

Ultraflexible organic photodetectors (OPDs) exhibiting high performance at near‐infrared wavelengths are developed, based on which a skin‐integrated multimodal system enabling time‐synchronized monitoring of photoelectric and biopotential signals is constructed. The sub‐20 µm‐thick system comprises OPDs, conductive bioelectrodes, and a hydrogel substrate. The seamless integration between the sensing components and the skin enables precise tracking of multiple vital signs with long‐term stability.

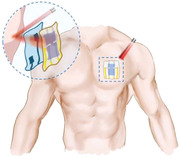

## 1 Introduction

Wearable electronic devices with a flexible form factor have unique advantages, including high conformability to the skin, high data extraction precision, and low motion artifacts,^[^ ^1^ ^]^ which will meet the demands for personalized health monitoring applications. As we are stepping into a new era of remote and decentralized patient care, wearable technologies, especially skin‐integrated electronics, will find enormous opportunities transforming from daily gadgets to clinical practices.^[^ ^2^ ^]^ Among variable physiological signals, vital signs, including the heartbeat rate (HR), respiration rate (RR), and blood pressure (BP), are important indicators of cardiovascular health conditions.^[^ ^3^ ^]^ Besides, blood oxygen saturation (SpO_2_), defined as the fraction of oxygen‐saturated hemoglobin relative to the total hemoglobin in the blood, is an essential indicator to detect hypoxia, the abnormally low oxygen levels in the human body. Wearable and skin‐conformable devices that allow real‐time and continuous monitoring of the signals mentioned above are particularly needed for home‐based care of patients with symptoms or mild forms of COVID‐19 infection, and aged groups living with chronic conditions. However, multichannel integration within an ultraflexible configuration confronts numerous challenges, mainly a lack of high‐performance and stable sensing components and an ideal design configuration that allows unconstrained movement and breathing of the skin.

Flexible photoplethysmography (PPG) sensors and pulse oximeters have been built by integrating flexible photodetectors and light‐emitting diodes, capable of extracting the pulse information from volumetric changes in the blood vessel in a real‐time and noninvasive manner.^[^ ^4^ ^]^ Near‐infrared (NIR) wavelengths penetrate deep into the skin and even probe subcutaneous blood volume variations. The stable penetration depths of the red‐NIR diagnostic window promote the measurement precision of SpO_2_ without harming the tissues.^[^ ^1a^ ^]^ Documented PPGs are mostly based on Si‐based photodetectors,^[^ ^5^ ^]^ which have certain limitations in their conformability to soft, wet, and dynamic skin. The resulting motion artifacts caused by the fluctuations of physical distance during the measurement further complexe the algorithm and limit the measurement precision.

Considerable research efforts were devoted to developing narrow bandgap organic semiconductors and facile solution processes, aiming at high‐performance NIR organic photodetectors (OPDs) with adjustable mechanical conformability and high performance.^[^ ^6^ ^]^ The development of narrow bandgap n‐type molecular semiconductors has demonstrated an effective strategy toward a potential substitute rival to silicon in photodetectors. In particular, 2,2′‐((2Z,2′Z)‐((5,5′‐(4,4‐bis(2‐ethylhexyl)‐4H‐cyclopenta[1,2‐b:5,4‐b′]dithiophene‐2,6‐diyl)bis(4‐((2‐ethylhexyl)oxy) thiophene‐5,2‐diyl))bis(methanylylidene))bis(5,6‐difluoro‐3‐oxo‐2,3‐dihydro‐1H‐indene‐2,1‐diylidene))dimalononitrile (COTIC‐4F), a non‐fullerene acceptor with an optical bandgap of ≈1.1 eV,^[^ ^7^ ^]^ absorbing light from 700 to 1100 nm, has exhibited photoresponsivity of 0.42 A W^−1^ at 995 nm.^[^ ^7b^ ^]^ However, recent progress on such material systems has not yet brought intrinsic flexibility and high electrical performance into full play. Documented OPDs having ultraflexible configuration and comparable photoresponsivity in the NIR range to silicon counterparts are rare, and their vast possibilities in skin‐integrated systems are to be explored.

The pulse signal provided by PPG is based on photoelectric volume change, while electrocardiogram (ECG) signals, the gold standard for measuring the heart rhythm and heartbeat rate, provide pulse‐related information based on bioelectricity. Conventional ECG systems are less appealing as daily gadgets given their bulkiness, inconvenience for long‐term wearing, and limited functions. The combination of wearable PPG and portable single‐lead ECG sensing components can improve the accuracy and reliability of pulse information and realize home‐based care in a user‐friendly fashion. Through the post‐processing of the two sets of signals, more health indicators, including blood pressure, can be obtained.^[^ ^8^ ^]^

Though flexible ECG electrodes or PPG sensors have been developed individually,^[^ ^9^ ^]^ building a reliable skin‐integrated PPG‐ECG integrated system is challenging. First, commercial ECG electrodes, such as Ag/AgCl gel electrodes or other metal‐based dry electrodes, are hard to couple steadily with ultraflexible photodetectors because of their bulky shape and mechanical mismatch. Second, individual sensing components should have high performance and long‐term working stability. Third, forming intimate and comfortable contact with the skin without causing dermatological irritation over a long period is a common challenge. Most skin‐integrated electronics^[^ ^1^, ^9^, ^10^ ^]^ need an additional adhesion layer to strengthen the interfacial toughness,^[^ ^11^ ^]^ increasing the risks of prohibiting the free “breathing” of skin. Besides, loosened adhesion and device contamination due to skin secretions are usually inevitable, resulting in the deterioration of optical transmittance and electrical performance. In short, a skin‐integrated patch capable of precise monitoring of photoelectric and biopotential signals requires a combination of high‐performance photodetectors and conductive electrodes, all together mounted in a soft substrate that interfaces directly with the skin without constraining its movement or breathing.

This study demonstrates a soft and compact patch that can monitor multiple vital signs—HR, RR, SpO_2_, and BP, exhibiting unprecedented mechanical compliance and skin compatibility. The essential components, that is, ultraflexible NIR OPDs, exhibit a photoresponsivity of 0.53 A W^−1^ (940 nm, −1 V reverse bias) and shot‐noise‐limited specific detectivity of 3.4 × 10^13^ Jones, cut‐off response frequency beyond 1 MHz at −3 dB, comparable with the performance of rigid Si counterparts. The flexible PPG sensor demonstrates a high signal‐to‐noise ratio (SNR) and stable peak‐to‐peak amplitude under hypoxic and hypoperfusion conditions, outperforming commercial finger pulse oximeters, and guaranteeing precise extraction of SpO_2_ in a dynamic working condition. We further integrated ultraflexible OPDs with conductive polymer electrodes by facile coupling via an ultrathin hydrogel interface.^[^ ^12^ ^]^ The hydrogel‐based electrodes exhibit a conductivity of >660 S cm^−1^ and a low skin‐contact impedance of 15 kΩ cm^2^ at 100 Hz, enabling high‐quality detection of electrophysiological signals. The integrated PPG‐ECG patch with a total thickness below 20 µm can be pasted or removed repeatedly without accelerating the degradation of electronic components. Various vital signs, including heartbeat rate, respiration rate, cuff‐less blood pressure, and arterial oxygen saturation, can be measured precisely, even under dynamic working conditions.

## 2 Results

### 2.1 Design of Skin‐Integrated Multimodal Sensing System

**Figure 1:**
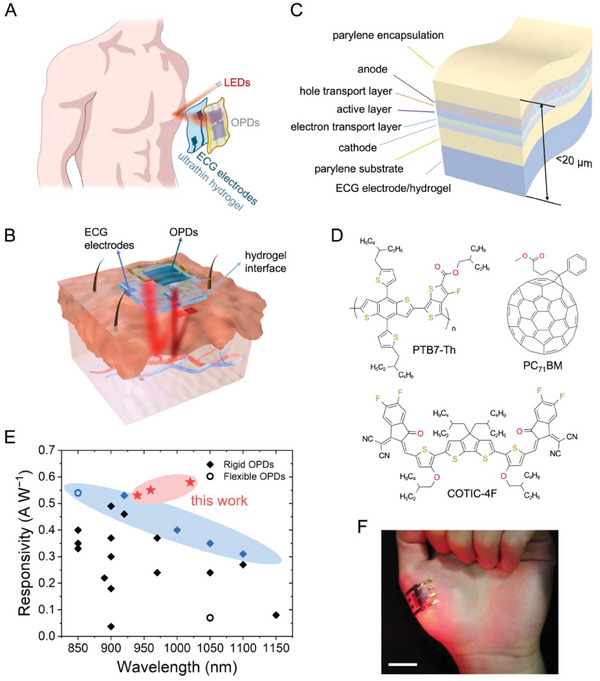
Design of the skin‐integrated PPG‐ECG system. A) Schematic of an ultraflexible integrated PPG‐ECG wearable patch. B) Schematic showing the cross‐section of an on‐skin patch consisting of ultraflexible OPDs and single‐lead ECG electrodes, assembled on two sides of an ultrathin hydrogel substrate that bridges the sensors with the skin directly. C) Schematic showing the device structure of ultraflexible OPDs, which are laminated on top of an ultrathin hydrogel film with pre‐patterned ECG electrodes on the other side. The overall thickness of the flexible sensing system is below 20 µm. D) Chemical structure of the donor polymer (PTB7‐Th), the non‐fullerene acceptor (COTIC‐4F), and the additive PC_71_BM adopted in the active layer of OPDs. E) Summary of the photoresponsivity from recently reported high‐performance NIR OPDs. The highest documented values of photoresponsivity in the NIR (850–1150 nm) region are marked in blue.^[^ ^9^, ^15^, ^24^, ^45^ ^]^ Solid rhombuses and hollow circles denote rigid and flexible devices, respectively. For detailed information, please refer to Table S1, Supporting Information. The photoresponsivity of ultraflexible OPDs developed in this work under 940, 960, and 1020 nm illumination (−1 V reverse bias) are highlighted in red stars. F) Photograph showing 4 µm‐thick freestanding OPDs adhered on the palm with high conformability. Scale bar, 2 cm.

The design layout and cross‐sectional schematic of the skin‐integrated PPG‐ECG patch is illustrated in **Figure** **1**A,B. Freestanding OPDs were encapsulated with parylene and then laminated on an ultrathin hydrogel substrate with ECG electrodes pre‐patterned on the other side (fabrication details given in Figure S1, Supporting Information). The hydrogel's high transparency, tailored interfacial strength, and ultrathin nature make it an ideal interface to couple ultraflexible PPG and ECG sensors and to integrate with human skin seamlessly.^[^ ^1^, ^13^ ^]^

A pulse oximeter based on ultraflexible photodetectors allows detecting the concentration of oxyhemoglobin (HbO_2_) and deoxyhemoglobin (Hb) within flowing blood, and subsequent calculation of SpO_2_ following: ${\text{SpO}}_{2}=\frac{{C_{\mathrm{HbO}}}_{2}}{{C_{\mathrm{HbO}}}_{2}+C_{\text{Hb}}}$, where *c* stands for the concentration. We use red (660 nm) and NIR (940 nm) illumination so that at 660 nm, Hb has a higher molar absorptivity than HbO_2_, and at 940 nm, HbO_2_ has a higher molar extinction coefficient, and the absorption coefficient ratio, that is, $\varepsilon_{\mathrm{Hb}}/\varepsilon_{\mathrm{Hb}O_{2}}$, is the most significant in the chosen spectrum regions (Figure S2, Supporting Information).^[^ ^9^, ^14^ ^]^ We adopt an inverted OPD structure (Figure 1C) that generally exhibits high photocurrent with improved stability,^[^ ^15^ ^]^ and a third‐phase additive strategy (chemical structures presented in Figure 1D) to improve the photoelectric conversion efficiency.^[^ ^16^ ^]^ The photoresponsivity of our ultraflexible OPDs under NIR light illumination reaches 0.53 A W^−1^ at 940 nm, and 0.55 and 0.58 A W^−1^ at 960 and 1020 nm, respectively, under a reverse bias of −1 V. The superior performance outstands among all documented NIR OPDs (Figure 1E).

Ultraflexible OPDs were fabricated on 1.5 µm‐thick parylene substrates (schematic in Figure 1C), with a total thickness below 4 µm. To minimize the strain applied to relatively brittle electrodes and active layer,^[^^]^ the thickness of the parylene substrate and passivation layer was determined following the equation that defines the position of the neutral mechanical plane, *b*

(1) $$b=\frac{\sum_{i=1}^{n}{\bar{E}}_{i}h_{i}\left[\left(\sum_{j=1}^{i}h_{j}\right)-\frac{h_{i}}{2}\right]}{\sum_{i=1}^{n}{\bar{E}}_{i}h_{i}}$$

where *E*_i_ and *h*_i_ denote Young's moduli and the thickness of each layer. As shown in Figure 1F, the flexible photodetector complies well with the curved and soft surface of the palm. The overall thickness of the PPG‐ECG integrated patch is below 20 µm (Figure 1C), guaranteeing high conformability to the skin texture,^[^ ^12^ ^]^ which will be explicitly addressed in a later part.

### 2.2 Ultraflexible High‐Performance Near‐Infrared OPDs

**Figure 2:**
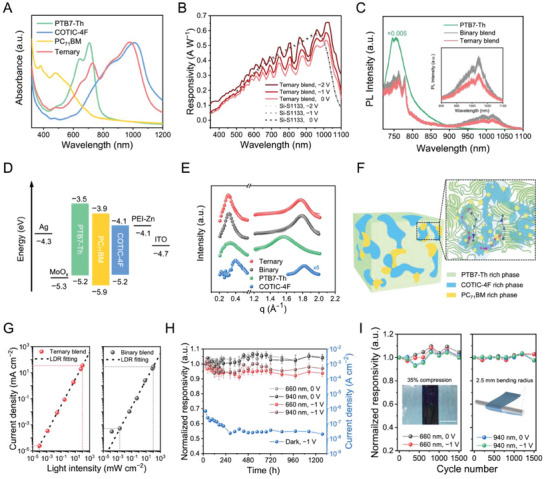
Ultraflexible high‐performance OPDs. A) UV–vis‐NIR absorption spectra of pristine PTB7‐Th, COTIC‐4F, PC_71_BM films, and their ternary blend at a ratio of 1:1.3:0.2. B) Comparison of photoresponsivity of the ultraflexible, ternary‐blend‐based OPD (in red, solid lines), and a commercially available Si‐S1133 photodetector (in grey and black, short‐dashed lines) under different bias conditions. C)  Photoluminescence spectra of pristine PTB7‐Th, binary blend, and ternary blend films, excited by a 633 nm laser. The insert displays PL spectra in the NIR region, excited by a 785 nm laser. D) Energy level diagram of the OPD based on the PTB7‐Th:COTIC‐4F:PC_71_BM ternary blend. E) GIWAXS out‐of‐plane cut profiles (5°–20°) of the ternary blend, binary blend, PTB7‐Th, and COTIC‐4F, respectively. F) Schematic of the microstructure in the PTB7‐Th:COTIC‐4F:PC_71_BM ternary blend at different length scales. The zoom‐in schematic highlights crystallized phases of the donor and acceptors and shows exciton formation and the subsequent dissociation and charge transport paths. G) The linear dynamic range of ultraflexible OPDs based on the ternary blend (left) and binary blend (right), under 660 nm illumination at a reverse bias of −1 V. H) Long‐term stability evaluation of ultraflexible OPDs based on the PTB7‐Th:COTIC‐4F:PC_71_BM ternary blend. I) Mechanical durability of ultraflexible OPDs. The left panel displays normalized responsivity variation as functions of cycle count during cyclic compression‐stretch testing. The inset provides a photograph of an ultraflexible OPD subjected to 35% compression (scale bar: 2 mm). The right panel presents the normalized responsivity variation observed throughout bending cycles, accommodating up to 1500 cycles, with a specified bending radius of 2.5 mm.

We first attend to the high photoelectric performance of ultraflexible OPDs in the NIR range. The active layer consists of a ternary blend of the polymer donor poly[4,8bis(5‐(2‐ethylhexyl)thiophen‐2‐yl)benzo[1,2‐b;4,5‐b′]dithiophene‐2,6‐diyl‐alt‐(4‐(2‐ethy‐lhexyl)3‐fluorothieno[3,4‐b]thiophene‐)‐2‐carboxylate‐(2‐6‐diyl)] (PTB7‐Th), a non‐fullerene acceptor COTIC‐4F,^[^ ^7^ ^]^ which have their characteristic absorption covering the required detecting window of SpO_2_, that is, 660 and 940 nm (**Figure** **2**A), and [6,6]‐phenyl C_71_‐butyric acid methyl ester (PC_71_BM) as an additive. Documented OPDs based on the PTB7‐Th:COTIC‐4F binary blend often show a reduced external quantum efficiency (EQE) in the NIR range (800–1000 nm) compared with the red region (600–800 nm).^[^ ^7^, ^18^ ^]^ Our observations show that OPDs based on the PTB7‐Th:COTIC‐4F:PC_71_BM ternary blend exhibit improved EQE in the NIR region, compared with the PTB7‐Th:COTIC‐4F binary counterparts (Figure S3A,B, Supporting Information). With 940 nm light illumination, ultraflexible OPDs based on the ternary blend exhibit a photoresponsivity of 0.48 (zero bias) and 0.53 A W^−1^ (−1 V reverse bias), respectively. Under a reverse bias of −2 V, the responsivity has reached 0.57 A W^−1^ at 940 nm and exceeded 0.60 A W^−1^ in the ≈1000–1050 nm spectra range, with a peak value of 0.65 A W^−1^ observed at 1020 nm (Figure 2B), surpassing the commercial silicon photodiode (Hamamatsu S1133‐01). From the statistical analysis on 12 devices for each structure, the ultraflexible OPDs based on PTB7‐Th:COTIC‐4F:PC_71_BM ternary blend exhibit a responsivity of 0.52 ± 0.02 A W^−1^ at 940 nm (−1 V reverse bias), higher than 0.49 ± 0.01 A W^−1^ of the binary counterparts (Figure S3C, Supporting Information). Besides, the −3 dB cut‐off response frequency of the binary and ternary blend OPDs are 0.95 and 1.14 MHz, respectively, under 940 nm illumination (Figure S3D, Supporting Information). The higher bandwidth may be ascribed to an improved carrier transport pathway and fewer trap‐states that facilitate the current collection and enhance the frequency response.^[^ ^15b^ ^]^

Beyond the inherent qualities of the active materials, the architectural design of ultraflexible devices also profoundly influences their performance. An optical interference effect was discerned from the performance‐wavelength plot of the ultraflexible OPDs, as visualized in Figure 2B. To elucidate such a phenomenon, we explored the optical transmittance of various layers within a sequential stack. On the basis of 1 mm‐thick soda‐lime glass, which exhibits an average transmittance of ≈90% across a wavelength range of 350 to 1200 nm, we deposited indium tin oxide (ITO) and then PEI‐Zn thin film in sequence, no signs of optical interference were observed (Figure S4A, Supporting Information). In contrast, introducing parylene coatings at thicknesses of 1.5 and 3 µm on the soda‐lime glass resulted in a discernible optical interference pattern (Figure S4B, Supporting Information). Delaminating parylene films into a free‐standing state intensified the optical interference, exhibiting oscillations around a transmittance value of 90% (Figure S4C, Supporting Information). Predominantly sourced from the parylene substrate and encapsulation layers, this optical interference further impacts the overall device transmittance and optoelectronic performance. Film thickness notably influences the periodical oscillation between coherence enhancement and attenuation—an observation that aligns with existing literature.^[^ ^19^ ^]^ Upon the subsequent deposition of thin films, including SU‐8 (the planarization layer), ITO electrode, and the PEI‐Zn ETL, there is a recognizable shift in the peak and valley positions (Figure S4D,E, Supporting Information). The transmittance exhibits periodic fluctuations with variations in both the wavelength and thickness of the films, eliciting further implications on the device's performance. Although a comprehensive, quantitative explanation of the coherence phenomena in multi‐layered films, consisting of varying materials, may pose challenges, our preliminary findings suggest possibilities of designing narrowband photodetectors optimized for specific wavelengths.

To gain insights into the impact of the additive PC_71_BM on the device performance, we conducted steady‐state photoluminescence (PL) spectrum measurement.^[^ ^20^, ^21^ ^]^ As shown in Figure 2C, the emission peak at 760 nm and the broad peak at ≈1000 nm can be attributed to PTB7‐Th and COTIC‐4F, respectively. While both the binary and ternary blends exhibit significant PL quenching compared to the pure PTB7‐Th, the magnitude of PL quenching is notably greater in the ternary blends. This observation suggests a more robust and efficient charge transfer (CT) process in the ternary system, as compared to the binary one.

To further understand the improved EQE with ternary blend, we begin with the definition which is generally given by the equation^[^ ^22^ ^]^

(2) $$EQE\;\left(\lambda \right)=\eta_{\mathrm{abs}}\;\left(\lambda \right)\eta_{\mathrm{gen}}\eta_{\mathrm{coll}}$$

where η_abs_ denotes the light absorption of the photo‐active layer, η_gen_ is the charge carrier generation quantum yield via exciton and CT state dissociation, and η_coll_ is the photo‐generated charge carrier collection efficiency. First, the ternary blend tends to produce more D:A interfaces, thus enhancing the absorption of CT states at a longer wavelength.^[^ ^23^ ^]^ Second, the introduction of PC_71_BM results in a cascade energy level alignment^[^ ^7^, ^16^, ^24^ ^]^ (Figure 2D) and increases the exciton dissociation efficiency at the D:A interfaces.^[^ ^25^ ^]^ Third, PC_71_BM leads to higher crystallinity of the acceptor phase, further promoting charge transport percolation pathways, as evidenced by grazing‐incidence wide‐angle X‐ray scattering (GIWAXS) measurements detailed below.

For bare COTIC‐4F films spin‐coated from CB and CB: CN (98:2) mixed solvent, an edge‐on to a face‐on orientation transition appears with adding CN in the solvent (Figure S5A,B, Supporting Information). This orientation transition benefits the out‐of‐plane (OOP) charge transport along the π–π stacking direction in an OPD configuration. Therefore, we used the CB:CN (98:2) mixed solvent to prepare blended films. Figures S5C and S5D, Supporting Information, show the 2D GIWAXS data of PTB7‐Th:COTIC‐4F (binary) and PTB7‐Th:COTIC‐4F:PC_71_BM (ternary) films, respectively. Cake cut profiles and the corresponding fit results in the OOP direction are presented in Figure 2E. The binary film shows a π–π stacking peak located at 1.77 Å^−1^ with a π–π distance of 3.55 Å in the OOP direction, which is attributed to a face‐on orientation of the COTIC‐4F crystalline phase. In the in‐plane (IP) direction, the signal at 0.31 Å^−1^ (20.27 Å) is assigned to the lamellar scattering of either PTB7‐Th or COTIC‐4F. After adding PC_71_BM to the binary system, the lamellar stacking characteristics basically remain unchanged compared to the binary film along the IP direction. Only a slight peak shift to 1.76 Å^−1^ occurs, corresponding to a less compact π–π stacking distance of 3.57 Å in the OOP direction. Therefore, introducing PC_71_BM would not alter the stacking property or crystallographic orientation in the bulk heterojunction.

The relevant π–π stacking data are listed in Tables S2 and Table S3, Supporting Information. Given that the (010) peak is closer to the acceptor side, it is reasonable to speculate that its distribution is likely attributable to COTIC‐4F, rather than PTB7‐Th.^[^ ^26^ ^]^ Domain size of the crystalline phases can be estimated by the crystal coherence length (CCL)^[^ ^27^ ^]^

(3) $$CCL=\frac{2\pi k}{FWHM}$$

where *k* is a shape factor, typically defined as 0.9. FWHM is the full width at half maximum of the diffraction peak. As detailed in Table S2, Supporting Information, the CCL associated with the π–π stacking ((010) peak in OOP direction) in the binary and ternary films were calculated as 25.70 and 28.27 Å, respectively. The slightly enhanced CCL indicates that the introduction of PC_71_BM potentially leads to higher crystallinity of the acceptor phase, which is favorable for efficient exciton dissociation and promotes charge transport percolation pathways (proposed microstructure shown in Figure 2F). With the above analysis, we suggest that the ternary blend might provide more exciton dissociation and transport sites by potentially enhancing the crystallinity of the acceptor phase.^[^ ^23^, ^28^ ^]^

Notably, as dark current and responsivity directly affect the SNR, a conspicuous dark current will complicate subsequent signal processing and hinder the readability of the output signal. Besides, mitigating dark current is of paramount importance to obtain high‐performance OPDs with desired specific detectivity (*D* ^*^) and linear dynamic range (LDR).^[^ ^15^, ^29^ ^]^ A detrimental effect of illumination‐history‐sensitive dark current was previously observed in devices with commonly used zinc oxide (ZnO) electron transport layer (ETL), due to its loss of charge selectivity after exposure to high‐energy photons.^[^ ^15c^ ^]^ With a flexible device configuration, the dark current increase might be more severe as cracks or interfacial deformation might appear under the mechanical strain.^[^ ^9^, ^24^, ^30^ ^]^

To minimize the dark current and maintain high working stability, we used PEI‐Zn (a chelated system between polyethyleneimine ethoxylated [PEIE] and Zn ions)^[^ ^24^, ^31^ ^]^ and spin‐coated the precursor twice, a “double‐ETL” strategy, to eliminate pinholes that potentially affect the device performance.^[^ ^15c^ ^]^ As shown in Figure S6A, Supporting Information, rigid OPDs having PEI‐Zn as the ETL show superior diode characteristics, that is, lower dark current under a negative bias and higher conductivity under a positive bias, compared with counterpart devices using ZnO or PEIE as the ETLs. In particular, devices based on the ternary blend with PEI‐Zn ETL exhibit a dark current density of 2.2 × 10^−10^ and 1.1 × 10^−7^ A cm^−2^ at 0 and −1 V bias, respectively, only one order of magnitude higher than the Si counterpart (Figure S6B, Supporting Information). Such dark current density values are among the lowest of documented flexible NIR OPDs.

The shot‐noise‐limited specific detectivity (*D*_sh_^*^) can be calculated from the responsivity (*R*) and the dark current density (*J*_d_), following the equation^[^ ^7b^ ^]^

(4) $${D_{\mathrm{sh}}}^{*}=\frac{R}{\sqrt{2qJ_{d}}}$$

where *q* is the elementary charge. The resulting *D*_sh_^*^ of ultraflexible OPDs is 3.4 × 10^13^ Jones and 4 × 10^13^ Jones at 940 and 1020 nm (zero bias), respectively, among the highest in all documented NIR OPDs, and comparable to the performance of Si counterpart photodetectors (Figure S6C and Table S1, Supporting Information).

Notably, the assumption of shot noise as the primary influence on noise current in dark conditions can potentially lead to an overestimation of specific detectivity, *D* ^*^.^[^ ^32^ ^]^ To accurately determine *D* ^*^, we measured the actual noise current of the ternary blend‐based devices, using PEI‐Zn and ZnO as ETL layers, respectively. As detailed in Figure S6D, Supporting Information, the noise current (*S*_n_) exhibits a gradual decline with the increase in frequency. In the low‐frequency region (<10 kHz), where the dominance of 1/f noise (flicker noise) is evident, the noise current variances of PEI‐Zn and ZnO‐based devices are marginal, while the silicon detectors exhibit a much lower noise output. Within the high‐frequency region (10–100 kHz), the noise current in PEI‐Zn‐based devices is markedly reduced, in comparison to their ZnO‐based counterparts, even lower than the Si counterpart devices in the range of (30–45 kHz and 70–100 kHz). Specifically, the noise current of the PEI‐Zn‐based devices records a value of 1.35 × 10^−12^ A Hz^−1/2^ at 10 kHz, which then drops to 1.91 × 10^−13^ A Hz^−1/2^ at 100 kHz. The frequency‐independent shot noise (*S*_sh_), on the other hand, can be estimated by the equation *S*_sh_ = (2*qI*_d_)^1/2^, where *I*_d_ is the dark current. Thus, the low noise current of PEI‐Zn‐based devices can be attributed to the suppressed shot noise.

The noise equivalent power (NEP) can be characterized by the noise current, which is expressed by

(5) $$NEP=\frac{i_{n}}{R\sqrt{B}}$$

where *R* is the responsivity, *B* is the bandwidth, and *i_n_* is the noise current. At the wavelength of 940 nm, setting the bandwidth *B* to 1 Hz, the PEI‐Zn‐based devices exhibit an NEP of 2.99 × 10^−12^ W Hz^−1/2^ at 10 kHz and 4.23 × 10^−13^ W Hz^−1/2^ at 100 kHz. Based on the actual noise current (*S*_n_), the specific detectivity (*D* ^*^) is defined by

(6) $$D^{*}=\frac{R\sqrt{A}}{S_{n}}$$

where *R* is the responsivity, and *A* is the active area. For the frequency of 100 kHz, PEI‐Zn‐based devices achieve a *D* ^*^ of 4.73 × 10^11^ Jones—an increment greater than an order of magnitude compared to ZnO‐based counterparts (3.75 × 10^10^ Jones). As depicted in Figure S6E, OPDs constructed with a ternary blend and PEI‐Zn as the ETL attain a *D* ^*^ in the range of ≈1.48 × 10^11^–5.46 × 10^11^ Jones, and surpassing 1.48 × 10^11^ Jones at a wavelength of 1100 nm. Under the same testing conditions, Si‐based photodetectors manage a *D* ^*^ of 2.05 × 10^11^ Jones but fail to reach 10^11^ at 1050 nm illumination.

Finally, LDR reflects the reliable detection range of a photodetector, and its definition is^[^ ^33^ ^]^

(7) $$LDR=20\;\log\frac{J_{\max}}{J_{\min}}=20\log\frac{L_{\max}}{L_{\min}}$$

where *J* and *L* represent the current density and light intensity, respectively. As the lower limit of LDR is determined by the dark current,^[^ ^34^ ^]^ the resulting LDR of ternary and binary blend‐based ultraflexible OPDs are >123 and >114 dB (under 660 nm illumination), respectively, further suggesting a superior performance of the PTB7‐Th:COTIC‐4F:PC_71_BM ternary‐blend OPDs (Figure 2G). The above results conclusively substantiate the outstanding performance of OPDs based on the ternary blend of PTB7‐Th:COTIC‐4F:PC_71_BM and having PEI‐Zn as the ETL layer.

The long‐term stability of ultraflexible devices is paramount for practical applications. After storage in ambient condition at room temperature (R.T., humidity of 20%) for 1272 h, freestanding ultraflexible OPDs (ternary blend, PEI‐Zn as the ETL) maintain high responsivity, that is, 0.34 and 0.52 A W^−1^ under the red (660 nm) and NIR (940 nm) light illumination (−1 V reverse bias), respectively, greater than 90% of the initial values (Figure 2H). Notably, we observed a decrease in the dark current density of ultraflexible OPDs, from 10^−7^ to 2.3 × 10^−8^ A cm^−2^ after shelf storage for 240 h, which then stabilized at 2 × 10^−8^ A cm^−2^ till 1272 h (−1 V reverse bias, blue trace in Figure 2H), while no discernable change was observed in photocurrent during the period recorded. We anticipate that the ternary blend undergoes subtle phase reorganization over time till a stabilized microstructure is formed, during which a great portion of trap states are filled, leading to a decrease in the dark current. The resulting *D*_sh_^*^ maintains up to 6.3 × 10^13^ Jones and 6.7 × 10^13^ Jones at 940 and 1020 nm (zero bias), respectively, after long‐term storage for 1272 h in ambient conditions (Figure S6F, Supporting Information).

Further, to evaluate the mechanical robustness of the ultraflexible OPDs, we transferred the 4 µm‐thick, freestanding devices onto a pre‐strained elastomeric substrate, specifically the 3 M very‐high‐bond (VHB) tape. Relaxing the pre‐stretched elastomer resulted in a 35% compressive strain applied to the OPDs atop (Figure 2I, left panel). Following 1500 compression‐stretch cycles, no significant performance degradation was detected. In another test configuration, the freestanding OPDs were repeatedly wrapped around a stainless‐steel rod with a bending angle of 120° and an approximate bending radius of 2.5 mm (Figure 2I, right panel). After 1500 bending cycles, no discernible deterioration in performance was observed. These results indicate a structurally stable and mechanically robust device performance under strain.

### 2.3 Skin‐Integrated PPG Sensor

**Figure 3:**
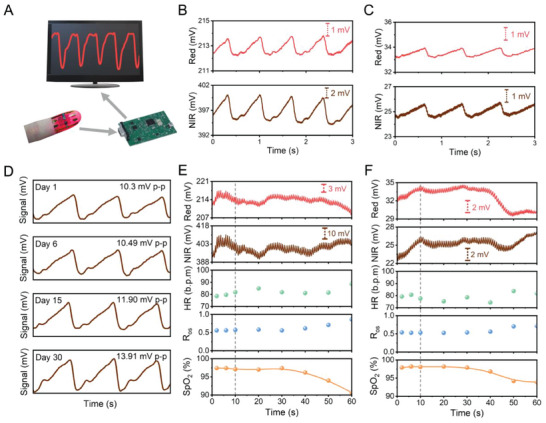
Skin‐integrated PPG sensor. A) Schematic of the PPG measurement setup. B,C) Comparison of the PPG signal obtained by B) our flexible sensor based on ultraflexible OPDs, and C) a commercial finger pulse oximeter. D) PPG signals recorded by an ultraflexible sensor within a storage period of 1 month. The device was stored in the ambient environment. Data were processed by band‐pass digital filtering. E,F) Data generated during the 1‐min breath‐holding experiment, including the pulse waves (red and brown), the heart rate (green), the calculated *R*_os_ (blue), and the fitted SpO_2_ values (orange) over time, using E) our flexible sensor and F) the commercial finger pulse oximeter. The subject remained still during the recording, and the breath‐holding began 10 s later. The volunteer is a healthy 31‐year‐old male from East Asia with no history of cardiovascular disease.

Ultraflexible OPDs having a high *R* and D* under red and NIR light, and rapid response speed, enable reliable PPG signal detection with low power consumption. Using a commercial data processing module (TI AFE4490EVM) integrated with 660 and 940 nm LEDs, we demonstrate a flexible PPG capable of high‐quality recording of pulse waves (testing configuration presented in **Figure** **3**A and Figure S7A, Supporting Information). Changes in the microvascular blood volume are translated into pulse waves.^[^ ^35^ ^]^ We compared signals recorded by our flexible PPG and a commercial finger pulse oximeter that integrates rigid Si photodetectors (both at zero bias), under the same LED illumination intensity (driving current of 10 mA) and using the same signal amplification method (100 kΩ trans‐impedance amplifier, TIA). As shown in Figure 3B, we obtained high‐quality PPG signals using our flexible system in a transmission mode. The peak‐to‐peak (p‐p) amplitude is ≈1.5 mV under 660 nm illumination, and ≈5.2 mV under 940 nm illumination. With the same volunteer and the measurement location, PPG signals obtained by the commercial finger pulse oximeter exhibited a lower p‐p amplitude, that is, ≈0.9 mV under 660 nm illumination, and ≈1.3 mV under 940 nm illumination (Figure 3C). The SNR of our flexible PPG sensor outperforms that of the rigid commercial counterpart, that is, 17.8 over 6.3. The high SNR is mainly attributed to the ultraflexible configuration that enables extreme mechanical compliance and excellent conformability to the skin surface, which leads to minimized light dissipation and motion artifacts.^[^ ^1h^ ^]^ Notably, the dicrotic notch (Figure S8, Supporting Information), an important indicator of heart and arterial health,^[^ ^4^, ^36^ ^]^ is clearly resolved in the PPG waves obtained by our flexible system, indicating the ability to capture both the systolic and diastolic phases. However, such details cannot be revealed in the recorded traces by the commercial rigid oximeter.

We evaluated the long‐term stability of our flexible PPG sensor. After storage in ambient conditions for up to 30 days, the p‐p amplitude did not show significant change (Figure 3D). The PPG wave obtained after device storage for 30 days still exhibits high quality with a dicrotic notch clearly visible. The excellent recording precision and stability underscore their potential for long‐term health monitoring applications.

To further evaluate the data acquisition quality in a dynamic working environment, we performed a nearly 1‐min breath‐holding experiment, proactively reducing the peripheral vascular oxygen saturation. During the breath‐holding, we recorded the real‐time SpO_2_ level using our flexible sensor and the rigid commercial finger pulse oximeter simultaneously. As shown in Figure 3E,F, the breath‐holding started 10 s after the recording began, and we observed the fitted SpO_2_ value dropping from 98% to 90% after holding breath for 50 s. The curve calibration and fitting details are described in the Supporting Information and Figure S7B, Supporting Information. Briefly, SpO_2_ values were calculated through the molar extinction coefficient (ε) and the ratio of the absorbances at the two different wavelengths (*R*_os_). *ε* can be extracted from Figure S2, Supporting Information, and *R*_os_ was determined by the ratio of pulse (AC) signal to static (DC) signal under 660/940 nm illumination. As the peak values and p‐p amplitudes are essential parameters in SpO_2_ extraction, a low noise level and high amplitude directly lead to high accuracy. The resulting SpO_2_ dropping trend is consistent with the data recorded by a commercial finger clip module. Notably, in the final 10 s of breath‐holding, the SNR and p‐p amplitude of the commercial rigid oximeter dropped to ≈2 and 0.6 mV, respectively, imposing challenges in accurately evaluating SpO_2_. The decline in signal quality is likely due to decreased blood perfusion during breath‐holding,^[^ ^37^ ^]^ which results in reduced p‐p amplitude and SNR. These factors lead to a high level of error in data processing,^[^ ^38^ ^]^ inevitable for the rigid oximeter module. In contrast, our flexible sensor demonstrates an SNR exceeding 14 and a p‐p amplitude of ≈4.5 mV during breath‐holding, suggesting higher sensitivity for accurate operation under dynamic and hypoxic conditions.

**Figure 4:**
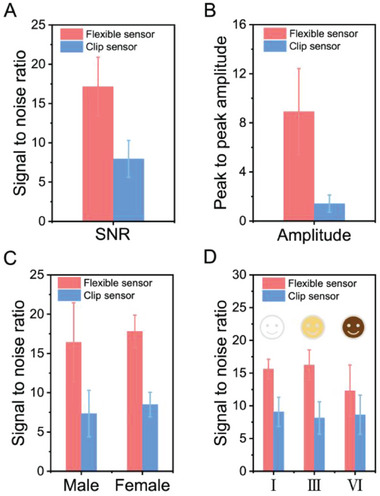
PPG signals derived from our ultraflexible PPG sensor (red bar) and a commercially available finger‐clip pulse oximeter (blue bar). A) The signal‐to‐noise ratio and B) peak‐to‐peak amplitude, both derived from a diverse sample of 21 volunteers. These volunteers, comprising 11 females and 10 males, represent varying backgrounds from East Asia, South Asia, and Southeast Asia. C) Statistic evaluation of the SNR recorded from a group of 11 female and another group of 10 male volunteers, respectively. Data depicted in panels (A–C) reflect measurements gathered from volunteers exhibiting the same Fitzpatrick skin tone, that is, Type III (golden honey). D) Evaluation of SNR values from a set of 10 volunteers with varying Fitzpatrick skin tone classifications, including Type I (pale white, three volunteers), Type III (golden honey, five volunteers), and Type VI (darkest brown, two volunteers), respectively. All tests were performed under 940 nm illumination.

PPG measurements can exhibit variations influenced by diverse skin tones and skin conditions.^[^ ^39^ ^]^ Consequently, the quality of PPG signals fluctuates from individual to individual. To investigate this, we enlisted 26 volunteers in total, encompassing diverse genders, geographic regions, and skin tones (refer to Table S4, Supporting Information, for detailed information), to partake in a PPG signal acquisition study comparing our ultraflexible sensor with a commercial finger‐clip pulse oximeter. First, we collected PPG signals from 21 volunteers from Asia sharing similar skin tones. Under 940 nm illumination, the statistical analysis of PPG signals derived from these volunteers demonstrated a superior performance by our flexible PPG sensor in terms of the SNR (**Figure** **4**A) and p‐p amplitude (Figure 4B), outperforming the rigid commercial counterpart. Specifically, the SNR is 17.15 ± 3.74 compared to 7.94 ± 2.35, and the p‐p amplitude registered at 8.9 ± 3.54 mV as against 1.42 ± 0.7 mV. We further examined the potential influence of gender on PPG signal quality. The initial cohort of 21 volunteers was segregated into male and female categories. As detailed in Figure 4C, the SNR of PPG signals recorded from both genders by our flexible sensors significantly exceeded those gathered with the commercial clip sensor. Specifically, for the 10 male volunteers, the SNR registered at 16.43 ± 5.01, markedly surpassing the 7.34 ± 2.96 recorded by the rigid clip sensor. Similarly, data from the 11 female volunteers yielded an SNR of 17.8 ± 2.07, well in excess of the 8.48 ± 1.56 by the commercial counterpart device.

Finally, we tested ten volunteers across different Fitzpatrick skin tone classifications^[^ ^40^ ^]^ including Type I (pale white), Type III (golden honey), and Type VI (darkest brown). Irrespective of skin type, our flexible PPG sensor persistently demonstrated superior SNR readings: 15.62 ± 1.50 over 9.07 ± 2.38 for Type I; 16.25 ± 2.28 over 8.14 ± 2.49 for Type III; and 12.31 ± 2.28 versus 8.62 ± 3.00 for Type VI (Figure 4D). These results underscore the remarkable adaptability and performance of our flexible sensor technology, transcending skin tone variations.

### 2.4 Skin‐Integrated PPG‐ECG Multimodal Sensing System

To ensure the performance of the integrated system, it is necessary to first verify the performance of hydrogel‐based bioelectrodes. The conductive polymer ink, a blend of poly(ethylenedioxythiophene):poly(styrenesulfonate) (PEDOT:PSS) with ethylene glycol (EG) and bis(trifluoromethane) sulfonimide lithium salt (LiTFSI), referred to as “PPEL” (PEDOT:PSS/EG/LiTFSI), was prepared following our established protocol.^[^ ^41^ ^]^ The PPEL electrodes were patterned on a 10 µm‐thick hydrogel substrate via drop‐casting through a PDMS mask (Figure S9A, Supporting Information). The ultrathin hydrogel is comprised of interpenetrating networks of polyacrylamide (PAAm) and sodium alginate, exhibiting an elastic modulus of 540 kPa.^[^ ^12^ ^]^ Following mild solvent evaporation and a post‐annealing process that enhances the interpenetration of the polymer network, we fabricated electrodes with a conductivity of 667 S cm^−1^, a sheet resistance of 1 Ω □^−1^, and a skin‐contact impedance of 15 kΩ cm^2^ at 100 Hz. Notably, our hydrogel‐based electrodes exhibit skin‐contact impedance that is only half that of commercial Ag/AgCl gel electrodes (Figure S9B, Supporting Information), suggesting their ability to effectively capture delicate bioelectrical signals in dynamic environments. Additionally, their ability to conform to irregular surfaces, biocompatibility, and high water‐vapor permeability^[^ ^12^ ^]^ make them attractive candidates for use in bio‐integrated systems, allowing natural movement and free breathing of the skin underneath.

The hydrogel‐based electrodes demonstrate exceptional operational stability, as evidenced by a mere 0.3% increase in sheet resistance following a 30‐day period of storage under ambient conditions (Figure S9C, Supporting Information). Our single‐lead ECG patch is intentionally designed to capture an ECG signal and registration comparable to that of lead II in a conventional 12‐lead ECG. The patch utilizes a dual‐electrode configuration to monitor potential differences, with an additional third electrode included to establish a low‐impedance return path that effectively reduces noise. This setup enables the flexible patch to couple with the skin directly and to screen both normal and pathological heart electrical activity.^[^ ^41^ ^]^

**Figure 5:**
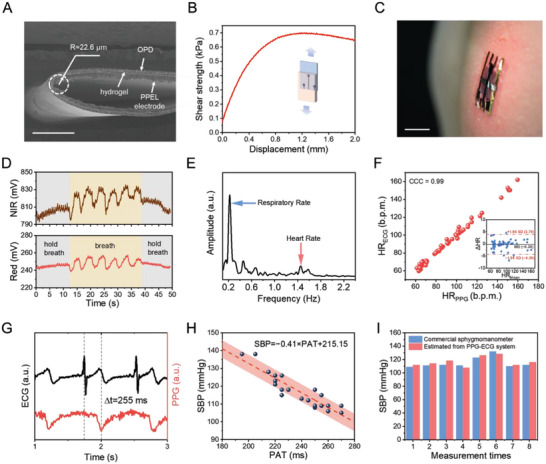
Skin‐integrated PPG‐ECG multimodal sensing system. A) An SEM image of the OPD/hydrogel/electrodes patch standalone, which exemplifies excellent mechanical compliance, capable of adapting to a bending radius down to 22.6 µm. Scale bar, 100 µm. B) Shear strength versus displacement as measured from a 10 µm‐thick hydrogel film with PPEL electrodes patterned on it. C) Photograph showing an integrated PPG‐ECG patch attached to the left chest of a male volunteer. Scale bar, 1 cm. D) PPG signals obtained using an integrated PPG‐ECG patch placed on the left chest of the same volunteer. The signals were obtained under red (660 nm) and NIR (940 nm) light illumination. The volunteer was instructed to perform natural breathing (light yellow area), as well as breath‐holding (gray area) while the signals were recorded. E) Fast Fourier transform of the recorded PPG signals in (D). F) Correlation plot of the simultaneous heart rate measurements acquired through the PPG module and the ECG electrodes. The inset depicts a Bland–Altman plot for heart rate data collected from three healthy adults using the integrated PPG‐ECG system. The data were recorded from volunteers #1, #13, and #21 (Table S4). G) Representative PPG‐ECG time‐synchronized signal obtained from an oscilloscope with a healthy male volunteer #21 from East Asia. The time interval Δ*t* denotes the pulse arrival time, the time difference between the R‐peak of ECG and valley regions of the PPG. H) The correlation between PAT obtained by our PPG‐ECG integrated system and SBP measured directly using a commercial sphygmomanometer (OMRON, J710). A linear fitting model was used to estimate blood pressure values. The margin of error (light red area) for the estimation model was set at ±5 mmHg, which was deemed acceptable based on scientific guidelines for non‐invasive blood pressure measurement. The data were recorded from volunteer #21. I) The estimated values of blood pressure using the derived PAT fitting model. Results are demonstrated in comparison with reference values read from a cuff‐based sphygmomanometer. The data were recorded from volunteer #21.

Finally, combining PPG with ECG electrode constituents a compelling and noninvasive tool to monitor multiple physiological parameters and directly assess cardiovascular health conditions.^[^ ^8^ ^]^ A PPG‐ECG integrated patch was built by laminating freestanding OPDs on an ultrathin hydrogel substrate that has pre‐patterned ECG electrodes on the other side (Figure S1, Supporting Information). The scanning electron microscopy (SEM) image in **Figure** **5**A reveals that the OPD/hydrogel/electrodes patch standalone can adapt to a bending radius as small as 22.6 µm, possessing a soft and compliant nature. When laminated onto a pre‐strained elastomer—specifically, VHB tape—and the elastomer's relaxation produces 100% compression on the patch, all layers consistently maintain close contact and effectively resist delamination, preventing the formation of interfacial air gaps. An SEM image illustrating the patch/VHB interface under compressive strain is depicted in Figure S9D, Supporting Information. These observations strongly suggest that the integrated sensing system possesses excellent mechanical compliance and stability.

The integrated patch was subsequently applied directly to the skin, positioning the ECG electrodes against it. The patch exhibited high conformability, seamlessly adapting to the intricate glyphic lines and subtle minutiae on the skin surface, as illustrated in the microscopic image in Figure S9E, Supporting Information. Further, the stable attachment of the sensing system on the skin is ensured by the hydrogel interfacial layer, with an interfacial strength of ≈0.4–0.6 N m^−1^ (Figure S10A, Supporting Information), and a shear strength of 0.70 kPa (Figure 5B), resulting in an average maximum shear force *P* of 0.52 N.

Notably, the ultrathin hydrogel substrate used in this study exhibits high optical transmittance, ensuring that the performance of OPDs and optical pulse oximeters remains unaltered. We conducted skin adhesion/peeling tests on 10 µm‐thick hydrogel films by subjecting them to more than five rounds of testing on the hand dorsum of five volunteers. The results indicate that the light transmittance values of all tested hydrogel samples remained above 97% within a wavelength range of 400–1100 nm. In contrast, commercial VHB tape (3 M VHB 4905) showed decreased light transmittance values of 95% at 660 nm and 92% at 940 nm (Figure S10B, Supporting Information). These findings provide a strong rationale for using ultrathin hydrogel substrate in optoelectronic systems.

We applied our integrated system to simultaneously record PPG and ECG signals from the left chest of three healthy East Asian male volunteers (illustration shown in Figure 5C). To extract the respiratory rate along with the PPG signal, we instructed the volunteers to control their breathing intentionally (Figure 5D). The Fourier transform of the PPG signals that were recorded from a volunteer has been depicted in Figure 5E. The high‐amplitude peaks observed at frequencies of 0.22 and 1.45 Hz correspond to the dominant respiration rate and heart rate, respectively. The individual who underwent testing had a respiratory rate of 13 breaths per minute and a heart rate of 87 beats per minute. To evaluate the validity of our measurements, we then conducted a concurrent assessment of heart rate using our integrated PPG‐ECG system and a commercial sphygmomanometer (OMRON, J710). Our results show a robust linear correlation across various measurement techniques, demonstrating a concordance correlation coefficient (CCC) exceeding 0.99 between the flexible PPG and ECG procedures, and 0.96 when comparing the results obtained by our flexible PPG and the commercial OMRON, respectively (Figure 5F and Figure S11A, Supporting Information). We further examined the accuracy of these correlations through a Bland–Altman plot analysis (inset in Figure 5F). The variation within ±1.96 standard deviations (SD) about the mean difference (MD) underscores an excellent agreement in HR derived via the three methods.

The combination of PPG and ECG signals can provide insights into cardiac output, as well as peripheral vascular resistance. Synchronized PPG and ECG signals further allow the estimation of systolic blood pressure (SBP). We attached the flexible PPG‐ECG integrated system to the fingertips of three healthy adult volunteers from East Asia. Concurrently, these individuals were equipped with a commercially available cuff‐based sphygmomanometer on their left arms. A representative PPG‐ECG synchronized signal captured from an oscilloscope is displayed in Figure 5G. The time interval denoted by Δ*t* represents the pulse arrival time (PAT), which has been shown to have a linear relationship with SBP. This relationship exists because changes in blood pressure can affect the flow rate of blood in the vessels, thereby influencing the PAT. Figure 5H demonstrates a linear correlation between PAT and SBP, modeled using the equation: SPB (mmHg) = −0.41 × PAT(ms) + 215.15, in which PAT is measured in milliseconds.

To evaluate the precision of the cuff‐less measurement configuration used in this study, we compared the estimated SBP values with those acquired using a commercial sphygmomanometer. The comparison was made in accordance with the ANSI (American National Standards Institute)/AAMI (Association for the Advancement of Medical Instrumentation) SP10‐1992 standard, which sets an acceptable error range of ±5 mmHg for non‐invasive blood pressure measurements (the red shadowed area in Figure 5H and Figure S11B,C, Supporting Information). Our analysis revealed that the estimated SBP values were within the acceptable error range, with a *p*‐value of <0.001 for each volunteer's measurements. A few data points displayed relatively large errors, primarily due to the lack of asynchronization between the two measurement systems. Specifically, the sphygmomanometer was activated half a minute later than the PPG‐ECG measurement, as tightening the cuff on the arm interferes with the measurement of PPG‐ECG signals from the hands. Subsequently, we employed the blood pressure estimation model to carry out eight additional measurements (Figure 5I). The discrepancies between the measured values and those recorded by the cuff sphygmomanometer remained within the 5 mmHg range, further suggesting the reliability of our flexible PPG‐ECG measurement configuration.

## 3 Conclusion

To summarize, the development of a skin‐integrated PPG‐ECG integrated system has great potential for personalized healthcare. We have developed OPDs that are ultraflexible and have long‐term stability, exhibiting high photoresponsivity under NIR light illumination. These OPDs show a photoresponsivity of 0.53 A W^−1^ under 940 nm (−1 V reverse bias) and a cut‐off response frequency beyond 1 MHz at −3 dB. After prolonged storage in ambient conditions for 1272 h, the photoresponsivity maintains over 90% of its initial values. Their shot‐noise‐limited specific detectivity of >10^13^ Jones is comparable to that of rigid Si counterparts. Our ultraflexible OPD‐based PPG outperforms its rigid commercial counterpart by having higher SNR and peak‐to‐peak amplitude, making it highly suited for precise measurement of SpO_2_, especially under dynamic and hypoxic conditions. Besides, we demonstrate soft hydrogel‐based electrodes exhibiting a conductivity of >660 S cm^−1^ and a low skin‐contact impedance of 15 kΩ cm^2^ at 100 Hz, enabling high‐quality detection of electrophysiological signals. The flexible PPG sensing module can be instantly integrated with the bioelectrodes and human skin via interfacial coupling of an ultrathin hydrogel film. The skin‐compatible sensing platform has a thickness of less than 20 µm and can be repeatedly applied or removed without any significant degradation of electronic components. We have demonstrated, for the first time, a skin‐integrated multimodal system capable of precisely and stably measuring various vital signs, including heartbeat rate, respiration rate, blood pressure in a cuff‐less manner, and arterial oxygen saturation, even under dynamic working conditions.

## 4 Experimental Section

### Chemicals

The chemicals used in this study were obtained from various suppliers and used without further purification. PTB7‐Th, COTIC‐4F, and PC_71_BM were purchased from Solarmer Material Inc. Zinc acetate dihydrate (Zn(Ac)_2_·2H_2_O), chlorobenzene (CB), 1‐chloronaphthalene (1‐CN), and 2‐methoxyethanol were obtained from J&K Scientific. PEIE solution (80% ethoxylated, 37 wt% in H_2_O) was purchased from Sigma‐Aldrich. PEDOT:PSS (PH1000, PEDOT:PSS ratio 1:2.5 by weight) was purchased from Clevios. Lithium bis(trifluoromethanesulfonyl)imide (LiTFSI, 99%) was obtained from Sigma‐Aldrich. EG (98%) was purchased from Macklin. The ITO target for magnetron sputtering, as well as gold, silver, and MoO_3_ source materials for thermal evaporation, were purchased from ZhongNuo Advanced Material.

### Fabrication of Ultraflexible OPDs

Soda‐lime glass with a size of 15 × 20 mm^2^ was taken as the rigid supporting substrate, and a fluorinated polymer (Delo D3, Shenzhen Sino‐fluorine) sacrificial layer was spin‐coated on top. A 1.5 µm‐thick parylene‐C film was deposited using an SCS parylene coater. A SU‐8 planarization layer was spin‐coated using a 1:1 SU‐8:developer solution, followed by post‐annealing at 180 °C inside a glove box to enhance the interfacial stability. The transparent ITO cathode with a thickness of 100 nm was deposited by a PVD‐75 series radio‐frequency magnetron sputtering system (K. J. Lesker Co.) under a 2 mTorr Argon (Ar) gas environment at room temperature. The power input was set to 100 W for 20 min. The ITO‐coated samples were then treated with O_2_ plasma under 100 W for 3 min. PEI‐Zn chelated film was subsequently deposited by spin‐coating the precursor solution (prepared by adding 0.3 g Zn(Ac)_2_·2H_2_O into 17.2 µL PEIE (37% in H_2_O) and 4 mL methoxyethanol with subsequent stirring for at least 3 h) and subsequent annealing at 180 °C for 10 min, repeated for twice, forming the “double‐layer” ETL. The active layer PTB7‐Th:COTIC‐4F:PC_71_BM (ternary system with a weight ratio of 1:1.2:0.3) or PTB7‐Th:COTIC‐4F (binary system with a weight ratio of 1:1.5) was deposited in glove box by spin‐coating the blend solution, with a mixed solvent of 20 mg mL^−1^ in chlorobenzene (98 vol%) and 1‐chloronaphthalene (2 vol%), at 1000 rpm for 60 s. The active layer thickness was optimized to ≈100 nm for both binary and ternary devices. The samples were then transferred to a high‐vacuum evaporator, where a 7.5 nm hole‐transport MoO*_x_* and 100 nm‐thick Ag anode were thermally deposited sequentially. Finally, the device was encapsulated with 1.5 µm‐thick parylene‐C film, and delaminated from the supporting glass, resulting in the ultraflexible OPDs ready for PPG measurement.

### Fabrication of the PPG‐ECG Integrated System

Ultrathin PAAm‐alginate hydrogel interfacial layer was prepared by a cold‐lamination method recently reported.^[^ ^12^ ^]^ The conductive polymer ink, a blend of PEDOT:PSS/EG/LiTFSI, referred to as “PPEL,” was prepared following the established protocol,^[^ ^41^ ^]^ that is, 50 mg EG and 5.9 mg LiTFSI powder added to 1 g PEDOT:PSS aqueous dispersion with a controlled solid concentration of 1.3 wt% (0.37 wt% PEDOT and 0.93 wt% PSS), followed by stirring for 15 min to ensure a complete intermixing. The PPEL‐based ECG electrodes were patterned on ultrathin hydrogel substrate via the drop‐casting method with the assistance of a soft PDMS mask. The solution casting was performed on a hot plate with a temperature set at 60 °C for mild solvent evaporation and then raised to 130 °C for 15 min to enhance the conductivity of the polymer network.^[^ ^42^ ^]^ Finally, the freestanding NIR OPDs were laminated on the blank side of the ultrathin and transparent hydrogel, with the optical path uncovered by the ECG electrode traces, forming the integrated PPG‐ECG patch.

### Optical Properties and Microstructure Characterization

The absorption and transmittance spectra were obtained on Shimadzu UV‐2600 equipped with an integrating sphere. Steady‐state PL spectra of films were measured by LabRAM HR Evolution (HORIBA Scientific). The films were excited by monochrome laser light sources with wavelengths of 633 and 785 nm, respectively. GIWAXS was performed at the P03 beamline, PETRA III, DESY in Hamburg (Germany), using an X‐ray beam energy of 11.83 keV (1.04 Å).^[^ ^43^ ^]^ The sample‐to‐detector distance (SDD) was set to 235 mm and the incident angle to 0.11°. The 2D GIWAXS transformation to q‐space and the cake cuts for the GIWAXS data were processed by the Matlab‐based package GIXSGUI.^[^ ^44^ ^]^ The surface morphology of an integrated PPG‐ECG system was observed via the Zeiss Sigma under an accelerating voltage of 5 kV. To remove the remaining water or organic solvent, all samples were lyophilized before being introduced to the SEM chamber.

### Evaluation of the OPD Performance

The EQE, responsivity, specific detectivity, noise current frequency spectra, and current–voltage (*J*–*V*) curves were recorded using an Enlitech PD‐QE system calibrated with standard Si and Ge photodetectors. LDR was measured under the illumination of a 660 nm‐LED light source equipped with neutral‐density filters, using a Keithley 2400 source meter. A network analyzer (Keysight N5227A) was used to measure the modulation bandwidth of the OPDs. The noise current was tested by an SR830 lock‐in amplifier (Stanford research systems) and an SR570 low‐noise current preamplifier (Stanford research systems). An analog front‐end evaluation board from Texas Instrument (AFE4490EVM) and its GUI software were used to process the acquired PPG signals and power the dual LEDs (660/940 nm, TE Connectivity).

### Electrical Properties of Hydrogel‐Based Electrodes

The sheet resistance of hydrogel‐based electrodes was measured using a four‐point geometry (RTS‐9) at three different locations, and the results were averaged for accuracy. Electrical conductivity (*σ*) was calculated using the formula $\sigma =\frac{1}{R_{s}\times t}$, where *t* represents the film thickness. To measure the electrode‐skin contact impedance, two electrodes with an effective area of 1 cm^2^ were placed on the anterior surface of a volunteer's forearm, with a distance of 5.5 cm between them. The electrodes were connected to an electrochemical workstation (CHI 760E), and impedance spectra were recorded in the frequency range from 10 to 10^4^ Hz.

### Evaluation of Mechanical Properties

Mechanical properties were evaluated using the uniaxial tensile test in an INSTRON 5966 with 100 N loading. Two ends of the sample were glued to acrylic plates and then connected to the fixture to prevent the device from slipping in the clamping positions.

The interfacial adhesion property was characterized by a 90° peel‐off test. The 1.5 µm‐thick parylene‐C film attached to a 10 µm‐thick hydrogel substrate was cut into a lateral size of 75 mm × 25 mm and interfaced with a piece of fresh porcine skin. Conductive polymer rectangles of 20 mm × 10 mm were pre‐patterned by drop casting on the hydrogel surface.

The shear strength was measured using the INSTRON 5966 with a pulling rate of 30 mm min^−1^. A 1.5 µm‐thick parylene‐C film coupled to a 10 µm‐thick hydrogel film was cut into 75 mm × 25 mm. The conductive polymer pattern was pre‐cast on one side of the hydrogel. All tests were performed with a controlled speed of 30 mm min^−1^ in ambient air at room temperature.

### PPG Signal Acquisition

For every volunteer, PPG signals were collected for over 40s duration, with volunteers remaining stationary during the recording period. SNR and peak‐to‐peak amplitude were calculated based on three stable pulse cycles. The measurements were conducted under zero bias. Tests involving SNR and peak‐to‐peak amplitude were performed under 940 nm illumination unless specifically mentioned otherwise.

### Signal Acquisition Using the Integrated PPG‐ECG System

RIGOL oscilloscope or the National Instruments NI‐9234 module was used, which was installed in the NI cDAQ‐9178 CompactDAQ chassis, to acquire the PPG and ECG signals synchronously. PAT was calculated from the time difference between the R peak of ECG and the trough of PPG, representing the time delay of the pulse wave originating from the aorta to the peripheral limb in each cardiac cycle. Assuming that the blood vessel was an elastic tube, a linear relationship between SBP and PAT can be established using empirical and theoretical models in the study of adult subjects, that is, SBP  =   − *a*(PAT) + *b* (where *a* and *b* denote the coefficient and a constant for linear regression calculation, respectively). An OMRON sphygmomanometer (J710) was used to simultaneously measure the SBP and heart rate, which were set as references to assess the data acquisition reliability of the skin‐integrated PPG‐ECG system. The measurements were conducted under zero bias. The three healthy volunteers, #1, #13, and #21 were all East Asian male volunteers, their ages were 25, 27, and 31, respectively.

### Statistical Analysis

The research methodology was meticulously screened and approved by the Ethical Committee of the Tsinghua Shenzhen International Graduate School at Tsinghua University (approval number 2023‐F035). Participants engaged in the study through an entirely voluntary selection processed from Tsinghua University Shenzhen International Graduate School and Peking University Shenzhen Graduate School.

Data assessment was conducted using device input generated by an unfiltered commercial analog front‐end or oscilloscope unless specifically mentioned otherwise. Collected data analysis was performed using Origin (2019b). Computed values were imparted as the mean ± standard deviation (SD), with accuracy upholding two decimal places. Variance in sample size (*n*) of OPDs, hydrogel‐based electrodes, and volunteers were considered.

The photodetector photoresponsivity was measured based on statistics from 12 devices of each structure. PPG readings were evaluated using SNR and p‐p amplitude, rooted in statistical results from 26 distinct individuals in total varying in gender and skin tone—the detail of the participants is provided in Table S4, Supporting Information.

For PPG‐ECG multimodal measurements, the fast Fourier transform was applied to PPG readings to derive breathing rate and heart rate. Statistical analyses were performed, determining the level of significance (*p*‐value) with an alpha threshold (*α*) set at 0.05. To establish a reliable linear fitting model for blood pressure approximation, 30 data sets collected via the PPG‐ECG multimodal system and a widely recognized commercial cuff‐based sphygmomanometer were combined. The latter acted as a trusted reference for blood pressure measurement comparisons. Out of these 30 data sets, 22 data sets were adopted for the model construction and validation, and the remaining 8 data sets to test the model's reliability. The accepted margin of error was ± 5 mmHg, following the precedent set by the ANSI/AAMI SP10‐1992 standard.

## Conflict of Interest

The authors declare no conflict of interest.

## Supporting information

Supporting Information

## Data Availability

The data that support the findings of this study are available in the supplementary material of this article.

## References

[1] a) G.‐H. Lee , H. Moon , H. Kim , G. H. Lee , W. Kwon , S. Yoo , D. Myung , S. H. Yun , Z. Bao , S. K. Hahn , Nat. Rev. Mater. 2020, 5, 149; 32728478 10.1038/s41578-019-0167-3 PMC7388681

[2] a) K. Bayoumy , M. Gaber , A. Elshafeey , O. Mhaimeed , E. H. Dineen , F. A. Marvel , S. S. Martin , E. D. Muse , M. P. Turakhia , K. G. Tarakji , M. B. Elshazly , Nat. Rev. Cardiol. 2021, 18, 581; 33664502 10.1038/s41569-021-00522-7 PMC7931503

[3] a) M. A. Cretikos , R. Bellomo , K. Hillman , J. Chen , S. Finfer , A. Flabouris , Med. J. Aust. 2008, 188, 657; 18513176 10.5694/j.1326-5377.2008.tb01825.x

[4] a) J. E. Sinex , Am. J. Emerg. Med. 1999, 17, 59; 9928703 10.1016/s0735-6757(99)90019-0

[5] H. Xu , L. Yin , C. Liu , X. Sheng , N. Zhao , Adv. Mater. 2018, 30, 1800156. 10.1002/adma.201800156 29806115

[6] a) P. C. Y. Chow , T. Someya , Adv. Mater. 2020, 32, 1902045; 10.1002/adma.201902045 31373081

[7] a) J. Lee , S.‐J. Ko , M. Seifrid , H. Lee , B. R. Luginbuhl , A. Karki , M. Ford , K. Rosenthal , K. Cho , T.‐Q. Nguyen , G. C. Bazan , Adv. Energy Mater. 2018, 8, 1801212;

[8] a) H. U. Chung , A. Y. Rwei , A. Hourlier‐Fargette , S. Xu , K. Lee , E. C. Dunne , Z. Xie , C. Liu , A. Carlini , D. H. Kim , D. Ryu , E. Kulikova , J. Cao , I. C. Odland , K. B. Fields , B. Hopkins , A. Banks , C. Ogle , D. Grande , J. B. Park , J. Kim , M. Irie , H. Jang , J. Lee , Y. Park , J. Kim , H. H. Jo , H. Hahm , R. Avila , Y. Xu , et al., Nat. Med. 2020, 26, 418; 32161411 10.1038/s41591-020-0792-9 PMC7315772

[9] a) E. O. Polat , G. Mercier , I. Nikitskiy , E. Puma , T. Galan , S. Gupta , M. Montagut , J. J. Piqueras , M. Bouwens , T. Durduran , G. Konstantatos , S. Goossens , F. Koppens , Sci. Adv. 2019, 5, eaaw7846; 31548984 10.1126/sciadv.aaw7846 PMC6744261

[10] T. Yokota , T. Nakamura , H. Kato , M. Mochizuki , M. Tada , M. Uchida , S. Lee , M. Koizumi , W. Yukita , A. Takimoto , T. Someya , Nat. Electron. 2020, 3, 113.

[11] J. Deng , H. Yuk , J. Wu , C. E. Varela , X. Chen , E. T. Roche , C. F. Guo , X. Zhao , Nat. Mater. 2021, 20, 229. 32989277 10.1038/s41563-020-00814-2

[12] S. Cheng , Z. Lou , L. Zhang , H. Guo , Z. Wang , C. Guo , K. Fukuda , S. Ma , G. Wang , T. Someya , H.‐M. Cheng , X. Xu , Adv. Mater. 2023, 35, 2206793. 10.1002/adma.202206793 36267034

[13] H. Yuk , B. Lu , X. Zhao , Chem. Soc. Rev. 2019, 48, 1642. 30474663 10.1039/c8cs00595h

[14] Y. Khan , D. Han , A. Pierre , J. Ting , X. Wang , C. M. Lochner , G. Bovo , N. Yaacobi‐Gross , C. Newsome , R. Wilson , A. C. Arias , Proc. Natl. Acad. Sci. U. S. A. 2018, 115, E11015. 30404911 10.1073/pnas.1813053115 PMC6255203

[15] a) K. H. Hendriks , W. Li , M. M. Wienk , R. A. J. Janssen , J. Am. Chem. Soc. 2014, 136, 12130; 25101518 10.1021/ja506265h

[16] a) Z. Wang , J. Ji , W. Lin , Y. Yao , K. Zheng , Z. Liang , Adv. Funct. Mater. 2020, 30, 2001564;

[17] a) D.‐H. Kim , J.‐H. Ahn , W. M. Choi , H.‐S. Kim , T. H. Kim , J. Song , Y. Y. Huang , Z. Liu , C. Lu , J. A. Rogers , Science 2008, 320, 507; 18369106 10.1126/science.1154367

[18] a) J. Vollbrecht , J. Lee , S.‐J. Ko , V. V. Brus , A. Karki , W. Le , M. Seifrid , M. J. Ford , K. Cho , G. C. Bazan , T.‐Q. Nguyen , J. Mater. Chem. C 2020, 8, 15175;

[19] J. Jean , A. Wang , V. Bulovic , Org. Electron. 2016, 31, 120.

[20] N. Gasparini , X. Jiao , T. Heumueller , D. Baran , G. J. Matt , S. Fladischer , E. Spiecker , H. Ade , C. J. Brabec , T. Ameri , Nat. Energy 2016, 1, 16118.

[21] X. Lai , S. Chen , X. Gu , H. Lai , Y. Wang , Y. Zhu , H. Wang , J. Qu , A. K. K. Kyaw , H. Xia , F. He , Nat. Commun. 2023, 14, 3571. 37322001 10.1038/s41467-023-39223-9 PMC10272153

[22] A. Armin , R. D. Jansen‐Van Vuuren , N. Kopidakis , P. L. Burn , P. Meredith , Nat. Commun. 2015, 6, 6343. 25721323 10.1038/ncomms7343

[23] Z. Tang , Z. Ma , A. Sánchez‐Díaz , S. Ullbrich , Y. Liu , B. Siegmund , A. Mischok , K. Leo , M. Campoy‐Quiles , W. Li , K. Vandewal , Adv. Mater. 2017, 29, 1702184. 10.1002/adma.201702184 28675522

[24] a) H. Guo , S. Saifi , K. Fukuda , H.‐M. Cheng , Z. Lou , X. Xu , Digital Signal Process. 2022, 125, 103145;

[25] F. Zhao , Y. Li , Z. Wang , Y. Yang , Z. Wang , G. He , J. Zhang , L. Jiang , T. Wang , Z. Wei , W. Ma , B. Li , A. Xia , Y. Li , C. Wang , Adv. Energy Mater. 2017, 7, 1602552.

[26] a) W. Huang , Z. Jiang , K. Fukuda , X. Jiao , C. R. Mcneill , T. Yokota , T. Someya , Joule 2020, 4, 128;

[27] D.‐M. Smilgies , J. Appl. Crystallogr. 2009, 42, 1030. 19953189 10.1107/S0021889809040126 PMC2779741

[28] A. A. Bakulin , A. Rao , V. G. Pavelyev , P. H. M. Van Loosdrecht , M. S. Pshenichnikov , D. Niedzialek , J. Cornil , D. Beljonne , R. H. Friend , Science 2012, 335, 1340. 22362882 10.1126/science.1217745

[29] a) W. Yang , W. Qiu , E. Georgitzikis , E. Simoen , J. Serron , J. Lee , I. Lieberman , D. Cheyns , P. Malinowski , J. Genoe , H. Chen , P. Heremans , ACS Appl. Mater. Interfaces 2021, 13, 16766; 33820414 10.1021/acsami.1c02080

[30] C. Fuentes‐Hernandez , W.‐F. Chou , T. M. Khan , L. Diniz , J. Lukens , F. A. Larrain , V. A. Rodriguez‐Toro , B. Kippelen , Science 2020, 370, 698. 33154137 10.1126/science.aba2624

[31] S. Xiong , K. Fukuda , S. Lee , K. Nakano , X. Dong , T. Yokota , K. Tajima , Y. Zhou , T. Someya , Adv. Sci. 2022, 9, 2105288. 10.1002/advs.202105288 PMC8922108 35064778

[32] a) Z. Xu , C. Sun , S. Min , Z. Ye , C. Zhao , J. Li , Z. Liu , Y. Liu , W.‐D. Li , M.‐C. Tang , Q. Song , H. Y. Fu , F. Kang , J. Li , Y. Shen , C. Yu , G. Wei , Small 2023, 2302072; 10.1002/smll.202302072 37431202

[33] Q. Li , Y. Guo , Y. Liu , Chem. Mater. 2019, 31, 6359.

[34] W. Li , Y. Xu , X. Meng , Z. Xiao , R. Li , Li Jiang , L. Cui , M. Zheng , C. Liu , L. Ding , Q. Lin , Adv. Funct. Mater. 2019, 29, 1808948.

[35] A. Kamisalic , I. Fister , M. Turkanovic , S. Karakatic , Sensors 2018, 18, 1714. 29799504 10.3390/s18061714 PMC6021794

[36] J. A. Chirinos , P. Segers , T. Hughes , R. Townsend , J. Am. Coll. Cardiol. 2019, 74, 1237. 31466622 10.1016/j.jacc.2019.07.012 PMC6719727

[37] J. Andersson , E. Schagatay , Undersea Hyperbaric Med. 1998, 25, 21. 9566083

[38] a) B. S. Kim , S. K. Yoo , IEEE Trans. Biomed. Eng. 2006, 53, 566; 16532785 10.1109/TBME.2005.869784

[39] B. Bent , B. A. Goldstein , W. A. Kibbe , J. P. Dunn , npj Digital Med. 2020, 3, 18. 10.1038/s41746-020-0226-6 PMC7010823 32047863

[40] T. B. Fitzpatrick , Arch. Dermatol. 1988, 124, 869. 3377516 10.1001/archderm.124.6.869

[41] B. Wei , Z. Wang , H. Guo , F. Xie , S. Cheng , Z. Lou , C. Zhou , H. Ji , M. Zhang , X. Wang , X. Jiao , S. Ma , H.‐M. Cheng , X. Xu , Cell Rep. Phys. Sci. 2023, 4, 101335.

[42] B. Lu , H. Yuk , S. Lin , N. Jian , K. Qu , J. Xu , X. Zhao , Nat. Commun. 2019, 10, 1043. 30837483 10.1038/s41467-019-09003-5 PMC6401010

[43] A. Buffet , A. Rothkirch , R. Döhrmann , V. Körstgens , M. M. Abul Kashem , J. Perlich , G. Herzog , M. Schwartzkopf , R. Gehrke , P. Müller‐Buschbaum , S V. Roth , J. Synchrotron Radiat. 2012, 19, 647. 22713902 10.1107/S0909049512016895 PMC3380660

[44] Z. Jiang , J. Appl. Crystallogr. 2015, 48, 917.

[45] a) Z. Zhong , F. Peng , L. Ying , G. Yu , F. Huang , Y. Cao , Sci. China Mater. 2021, 64, 2430;

