# Supplementary material for: Near‐Infrared Organic Photodetectors toward Skin‐Integrated Photoplethysmography‐Electrocardiography Multimodal Sensing System
Source: Adv Sci (Weinh). 2023 Nov 22;10(36):2304174. doi: 10.1002/advs.202304174 (PMC10754100; doi:10.1002/advs.202304174)
Supplement: Supplementary file 1 — Supporting Information [file ADVS-10-2304174-s001.pdf]

ADVANCED SCIENCE
Open Access

Supporting Information

for *Adv. Sci.*, DOI 10.1002/advs.202304174

Near-Infrared Organic Photodetectors toward Skin-Integrated Photoplethysmography-Electrocardiography Multimodal Sensing System

Zirui Lou, Jun Tao, Binbin Wei, Xinyu Jiang, Simin Cheng, Zehao Wang, Chao Qin, Rong Liang, Haotian Guo, Liping Zhu, Peter Müller-Buschbaum, Hui-Ming Cheng and Xiaomin Xu**

Supporting Information

Near-Infrared Organic Photodetectors toward Skin-Integrated Photoplethysmography-Electrocardiography Multimodal Sensing System

Zirui Lou, Jun Tao, Binbin Wei, Xinyu Jiang, Simin Cheng, Zehao Wang, Chao Qin, Rong Liang, Haotian Guo, Liping Zhu, Peter Müller-Buschbaum, Hui-Ming Cheng, Xiaomin Xu**

Dr. Zirui Lou, Jun Tao, Dr. Binin Wei, Simin Cheng, Zehao Wang, Haotian Guo
Prof. Xiaomin Xu
Shenzhen International Graduate School & Tsinghua-Berkeley Shenzhen Institute
Tsinghua University
Shenzhen 518055, China
E-mail: xu.xiaomin@sz.tsinghua.edu.cn

Dr. Zirui Lou
School of Advanced Materials,
Peking University Shenzhen Graduate School
Shenzhen, 518055 China

Xinyu Jiang, Prof. Peter Müller-Buschbaum
Lehrstuhl für Funktionelle Materialien, Physik Department
Technische Universität München
James-Franck-Str. 1, 85748, Garching, Germany

Chao Qin, Rong Liang, Prof. Liping Zhu
State Key Laboratory of Silicon and Advanced Semiconductor Materials,
School of Materials Science and Engineering
Zhejiang University
Hangzhou 310027, China

Prof. Peter Müller-Buschbaum
Heinz Maier-Leibnitz-Zentrum (MLZ)
Technische Universität München
Lichtenbergstr. 1, 85748, Garching, Germany

Prof. Hui-Ming Cheng
Institute of Technology for Carbon Neutrality & Faculty of Materials Science and Energy Engineering
Shenzhen Institute of Advanced Technology
Chinese Academy of Sciences
Shenzhen 518055, China

Prof. Hui-Ming Cheng
Shenyang National Laboratory for Materials Science
Institute of Metal Research, Chinese Academy of Sciences
Shenyang 110016, China
E-mail: hm.cheng@siat.ac.cn

Monitoring and extraction of vital signs

Extraction of SpO_2 values

PPG signals obtained under two wavelengths were used to calculate SpO_2 following the developed principle.[1] The calculation process involves simulating the propagation of light in tissue using the modified Beer-Lambert Law:

$$I(\lambda) = I_0(\lambda)e^{-\mu_a(\lambda)*d*DPF(\lambda)}$$

where $I(\lambda)$ is the measured light intensity, $I_0(\lambda)$ is the intensity of incident light, $\mu_a(\lambda)$ is the absorption coefficient of the induced tissue, d is the distance between the light emitter and the detector. $\mathrm{DPF}(\lambda)$ represents the differential path length factor (DPF), which takes the multiple scattering of light in tissue into account. The calculation formula for SpO_2, which incorporates the modified Beer-Lambert Law and principles of blood oxygen calculation, has been described in detail by Arias et al. in their publication,[1a] and will not be repeated here. To derive SpO_2, we use the following equation:

$$SpO_2(R'_{os}) = \frac{\varepsilon_{\lambda_{1,Hb}} - \varepsilon_{\lambda_{2,Hb}} R'_{os}}{\left(\varepsilon_{\lambda_{1,Hb}} - \varepsilon_{\lambda_{1,HbO_2}}\right) + (\varepsilon_{\lambda_{2,HbO_2}} - \varepsilon_{\lambda_{2,Hb}})R'_{os}}$$

where $\varepsilon_{\lambda,HbO_2}$ and $\varepsilon_{\lambda,Hb}$ represent the molar extinction coefficient of oxyhemoglobin (HbO_2) and deoxyhemoglobin (Hb) at each wavelength. $R'_{os} = \frac{R_{os}}{\frac{DPF_{\lambda_1}}{DPF_{\lambda_2}}}$, where $R_{os} = \frac{AC_{\lambda_1}/DC_{\lambda_1}}{AC_{\lambda_2}/DC_2}$ is the ratio of the pulse signal (AC) to the static signal (DC) at two wavelengths. The factor DPF_λ accounts for multiple scattering effect. By using the relationship between R'_{os} and SpO_2, we develop a calibration curve that enables precise calculation of SpO_2.

In our study, we used dual LEDs that emit light at wavelengths of 660 nm and 940 nm. At these specific wavelengths, the difference in the absorption coefficient ratio between Hb and HbO_2 is the most significant, and this ratio remains relatively stable around the target wavelength (**Figure S2**). This property enables accurate extraction of SpO_2.[2] The corresponding molar extinction coefficients were incorporated into our formula, and the value of DPF_{660}/ DPF_{940} was calculated using Monte Carlo simulation results from Kyriacou et al.,[3] using a value of 1.7. Subsequently, we derived our calibration curve (**Figure S7B**).

Our findings are consistent with the widely accepted empirical fitting equation, $SpO_2 = 110 - 25R_{os}$.[4] However, we observed a notable deviation from the empirical curve when we used the calibration curve without DPF coefficient correction. This deviation is not applicable to practical scenarios and can be attributed to the multiple scattering of light in human tissue,

which has a substantial impact on the results. Therefore, it is imperative to incorporate an absorption model for human tissue that adheres to the modified Beer-Lambert law.

Extraction of respiratory rate

Respiratory rate, which is an important vital sign in clinical settings, can be non-invasively obtained from PPG signals. The baseline of PPG signals exhibits periodic variations that are attributed to the cyclical action of breathing, making it an ideal signal for extracting respiratory rate. The PPG signals collected by flexible OPDs are analyzed using frequency domain technique, the Fast Fourier Transform (FFT) in this study, that extracts the respiratory rate in the low-frequency range, typically below 1 Hz.

Dicrotic notch

The dicrotic notch is a physiological event that occurs in the arterial pulse waveforms and corresponds to the closure of the aortic valve. This event marks the transition from systole, the contraction phase of the cardiac cycle, to diastole, the relaxation phase of the cycle. It is an important clinical marker for evaluating cardiac function and diagnosing various diseases. The presence of the dicrotic notch is considered and important clinical marker for evaluating cardiac function and diagnosing various diseases.

In **Figure S8**, a typical pulse waveform showing the dicrotic notch along with other relevant characteristics is presented.[5] This waveform can be used to assess various parameters related to cardiovascular health, such as heart rate, stroke volume, and blood pressure. By analyzing the timing, morphology, and amplitude of the dicrotic notch, healthcare professionals can gain insights into the underlying mechanisms of cardiac disorders and make appropriate treatment decisions. Overall, the observation of the dicrotic notch in pulse waveforms is an essential component of clinical practice and research in cardiology.

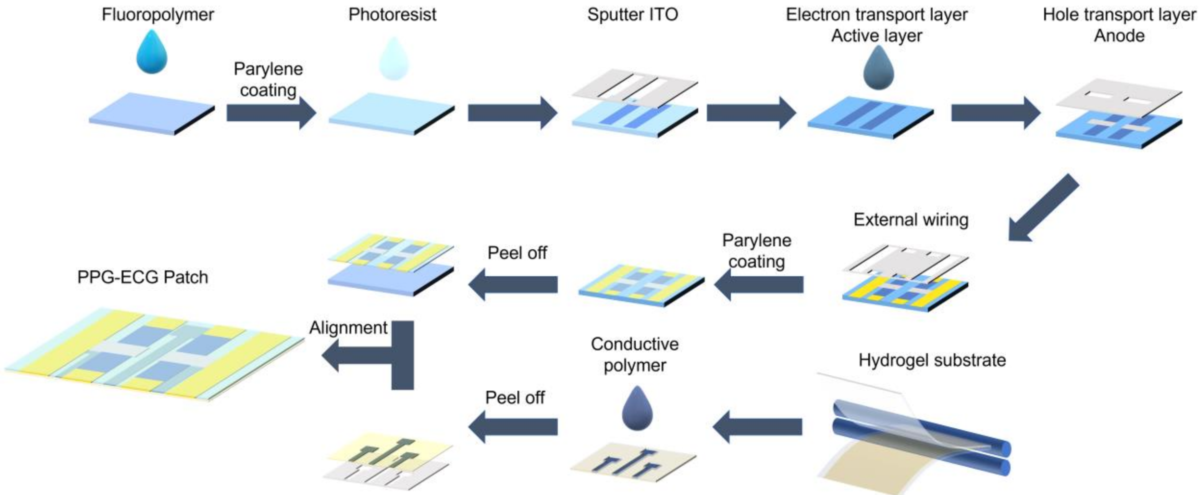

Figure S1. Schematic showing the fabrication procedure of the PPG-ECG patch.

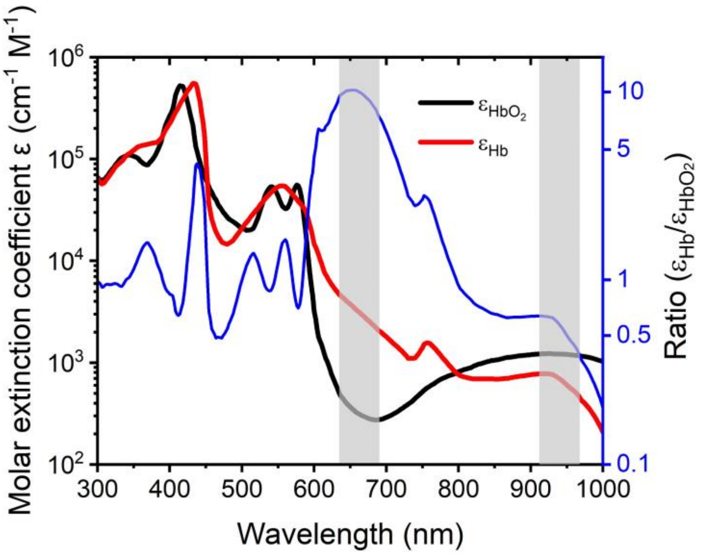

Figure S2. Molar extinction coefficient and the corresponding ratio of oxyhemoglobin to deoxyhemoglobin as a function of wavelength.

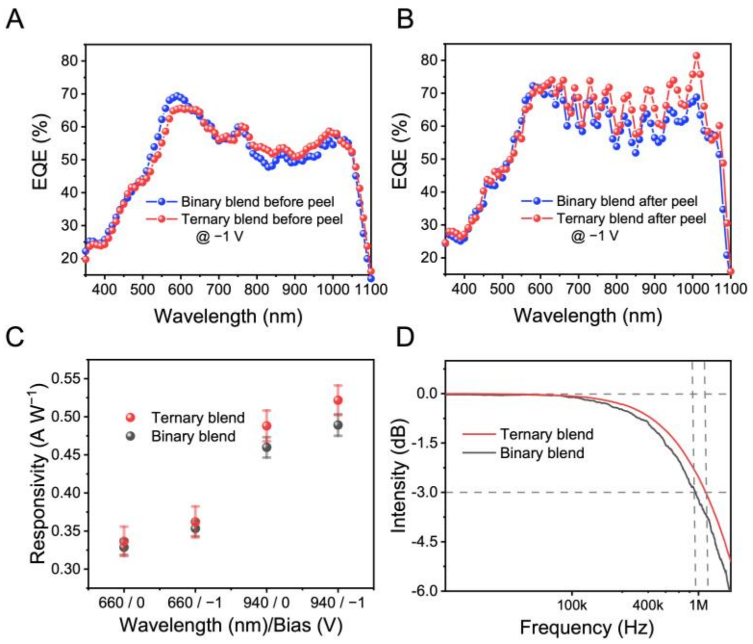

Figure S3. (A-B) EQE spectra of ultraflexible OPDs based on the binary blend (A) and the ternary blend (B), before and after the delamination from supporting glass. (C) Statistical results of the photoresponsivity taken from ultraflexible OPDs based on PTB7-Th:COTIC-4F binary blend (in gray) and the PTB7-Th:COTIC-4F:$PC_{71}BM$ ternary blend (in red), respectively. Statistics were performed based on 24 devices with an active area of 0.04 cm^2 for each structure. (D) The frequency response of ultraflexible OPDs based on the binary blend (in black) and ternary blend (in red) under 940 nm. The −3 dB cut-off response frequency of the binary and ternary blend-based OPDs are 0.95 MHz and 1.14 MHz, respectively.

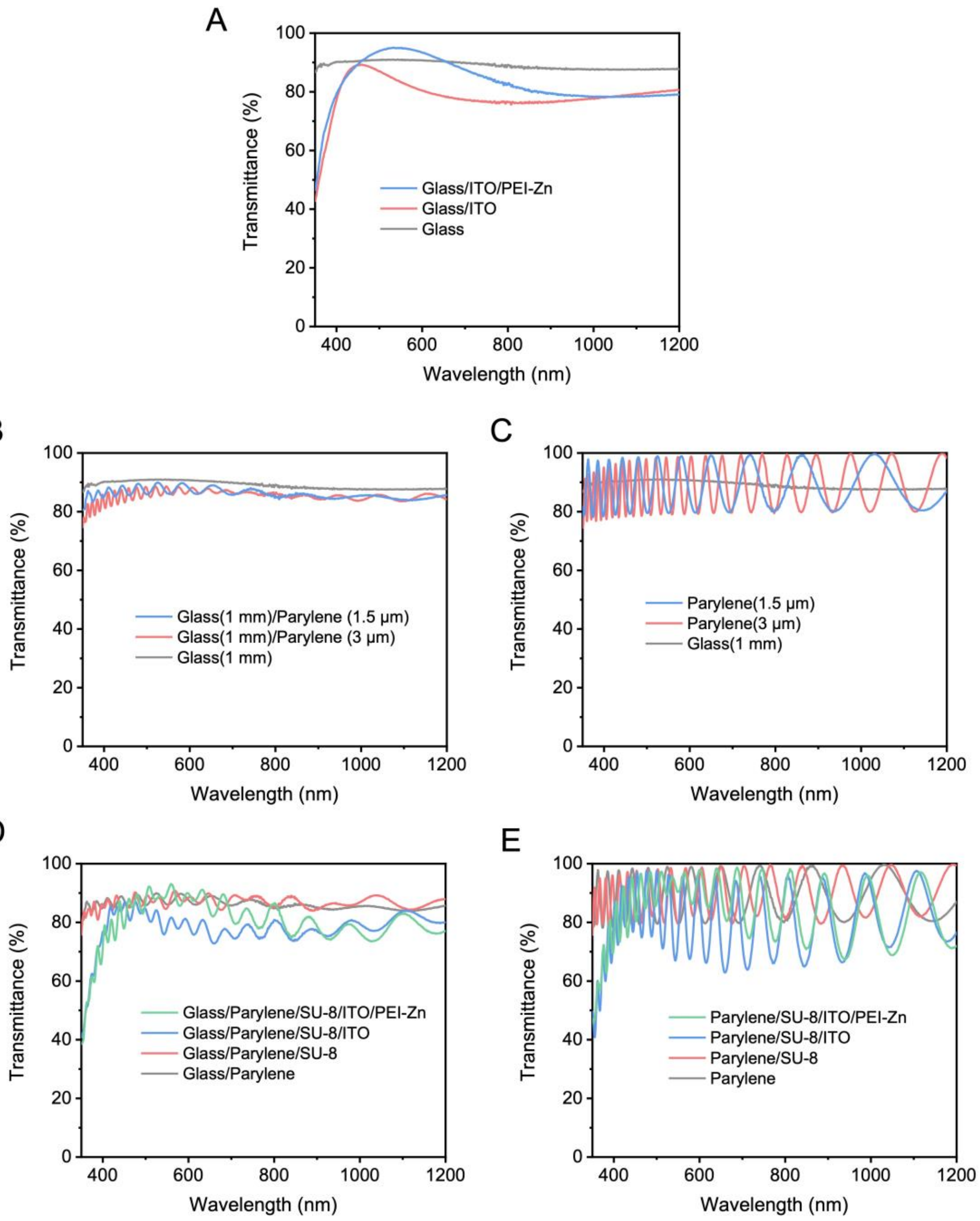

Figure S4. UV–vis transmission spectra. (A) Optical transmission spectra of soda-lime glass, soda-lime glass coated with sputtered ITO, and with PEI-Zn/ITO, respectively. (B) Optical transmission spectra of soda-lime glass, soda-lime glass coated with parylene film having thickness of 1.5 µm and 3 µm, respectively. (C) Optical transmission spectra of soda-lime glass, and 1.5 µm-thick and 3 µm-thick parylene films in a freestanding state. (D) Optical transmission spectra of soda-lime glass, soda-lime glass coated with parylene film, and further having SU-8, ITO, PEI-Zn in sequence. (E) Optical transmission spectra of freestanding parylene film, and parylene films further coated with SU-8, ITO, PEI-Zn in sequence.

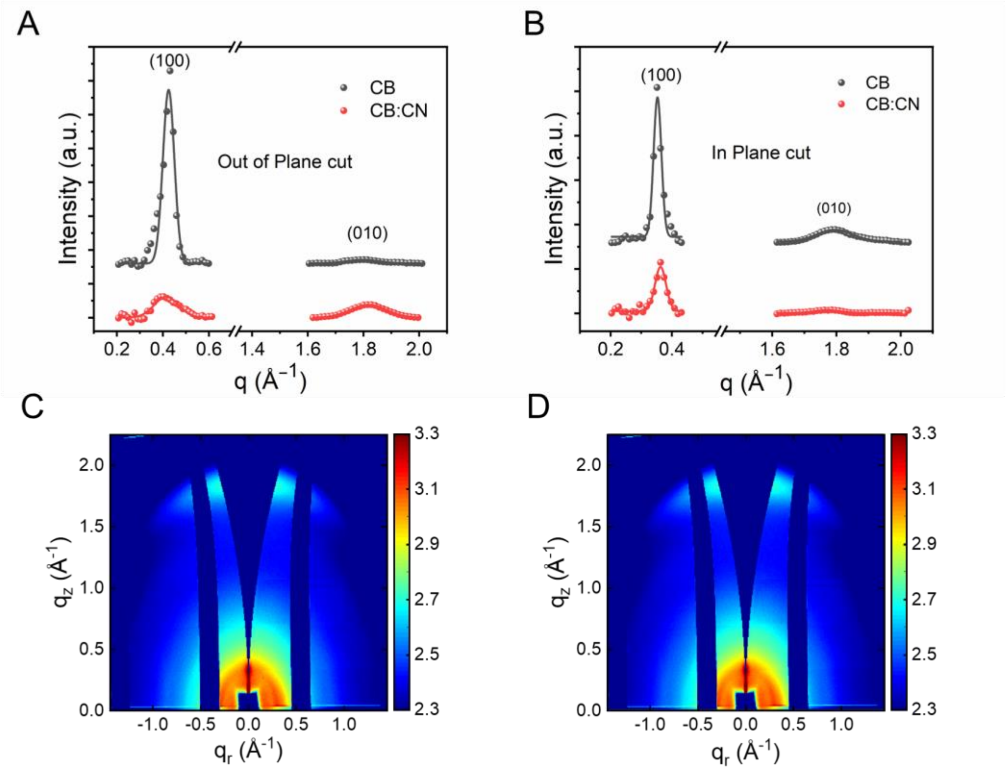

Figure S5. Cake cut profiles of COTIC-4F film with and without CN additive along the out-of-plane (A) and in-plane (B) direction. 2D GIWAXS data of the binary (C) and the ternary (D) blend films, respectively.

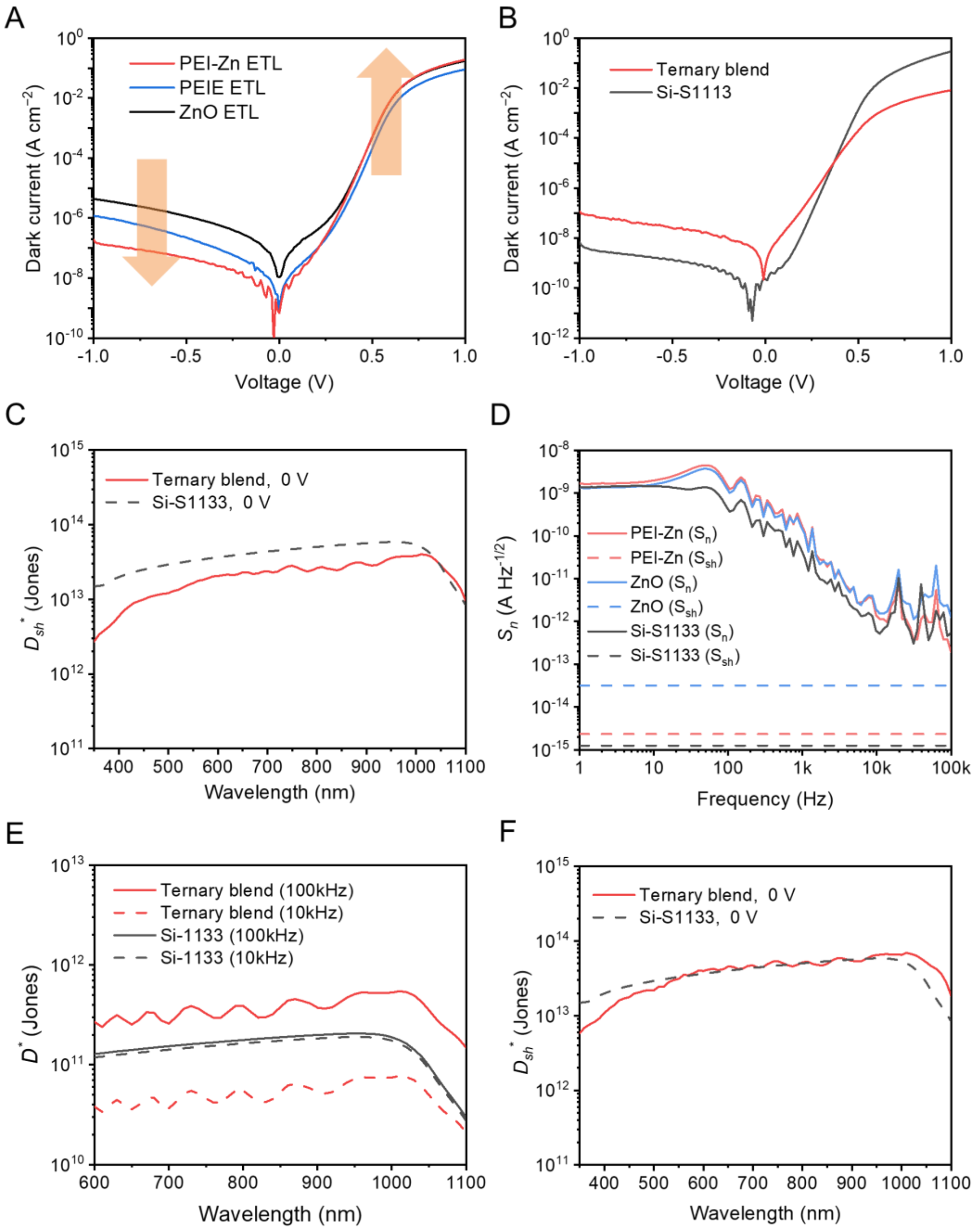

Figure S6. (A) Current-voltage (*J*-*V*) curves of rigid OPDs having PEI-Zn (red), PEIE (blue), and ZnO (black) as the electron transport layer, respectively, measured in dark. (B) *J*-*V* curves of an ultraflexible OPD based on PTB7-Th:COTIC-4F:PC_{71}BM ternary blend and with PEI-Zn electron transport layer, and a reference Si-S1133 photodetector (black). (C) Shot-noise-limited specific detectivity (D_{sh}^*) of an ultraflexible OPD based on the PTB7-Th:COTIC-4F:PC_{71}BM ternary blend, and a reference Si-S1133 rigid photodetector. The measurement was performed at zero bias. (D) Noise spectra for OPDs based on the ternary blend with either PEI-Zn (red) or ZnO (blue) as ETL, measured at zero bias, presented in comparison to a commercial Si-S1133 photodetector (black). The dashed lines are the shot noise limit for each device. (E) Specific detectivity (D^*) of a ternary blend-based ultraflexible OPD (red), and a reference Si-S1133 photodetector (black), evaluated based on the actual noise current at 10 kHz and 100 kHz respectively. (F) Stability evaluation of the D_{sh}^* for the ultraflexible OPD, in comparison with the same reference Si-S1133 photodetector. The freestanding OPD was stored in ambient condition (room temperature, controlled humidity of 20%) for up to 1272 h. All measurements were performed in ambient conditions.

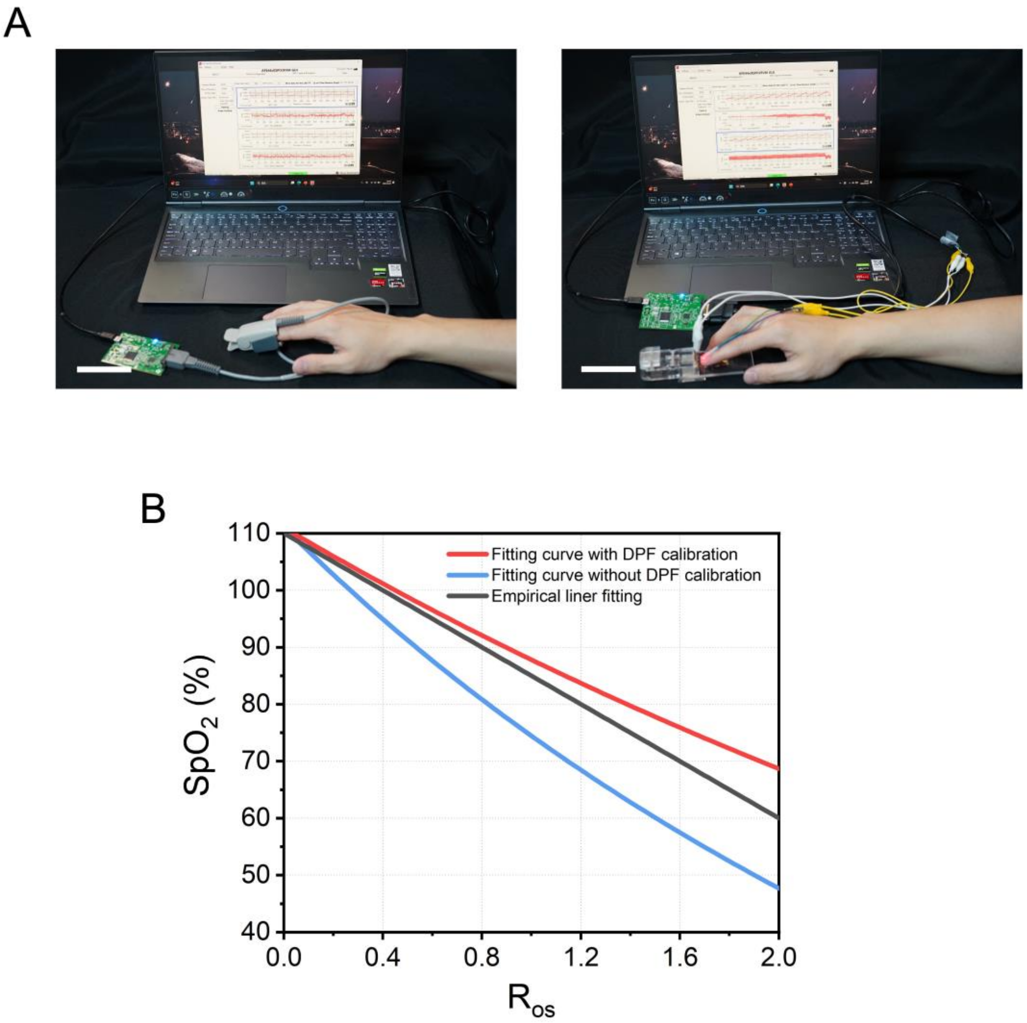

Figure S7. (A) Photographs of the PPG measurement setup using a commercial finger pulse oximeter under the transmission mode (left), an ultraflexible OPD under the transmission mode (middle). Scale bar, 4 cm. (B) The calibration curves for SpO_2. The curve fitted with DPF calibration was used in this work.

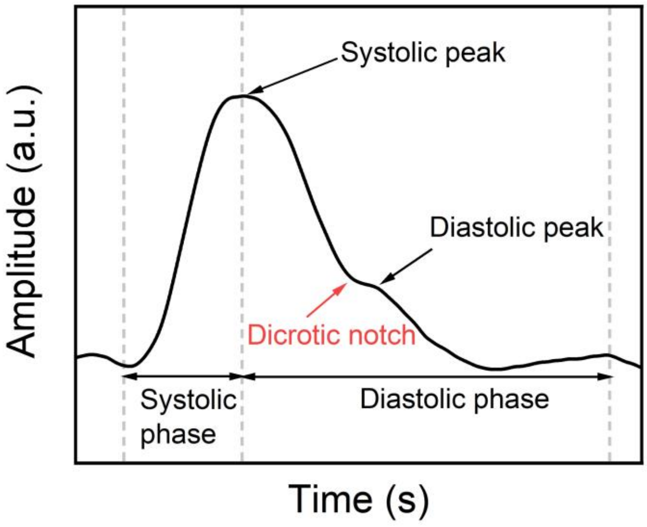

Figure S8. A typical PPG pulse wave showing the systolic and diastolic phases. The dicrotic notch is marked by the red arrow.

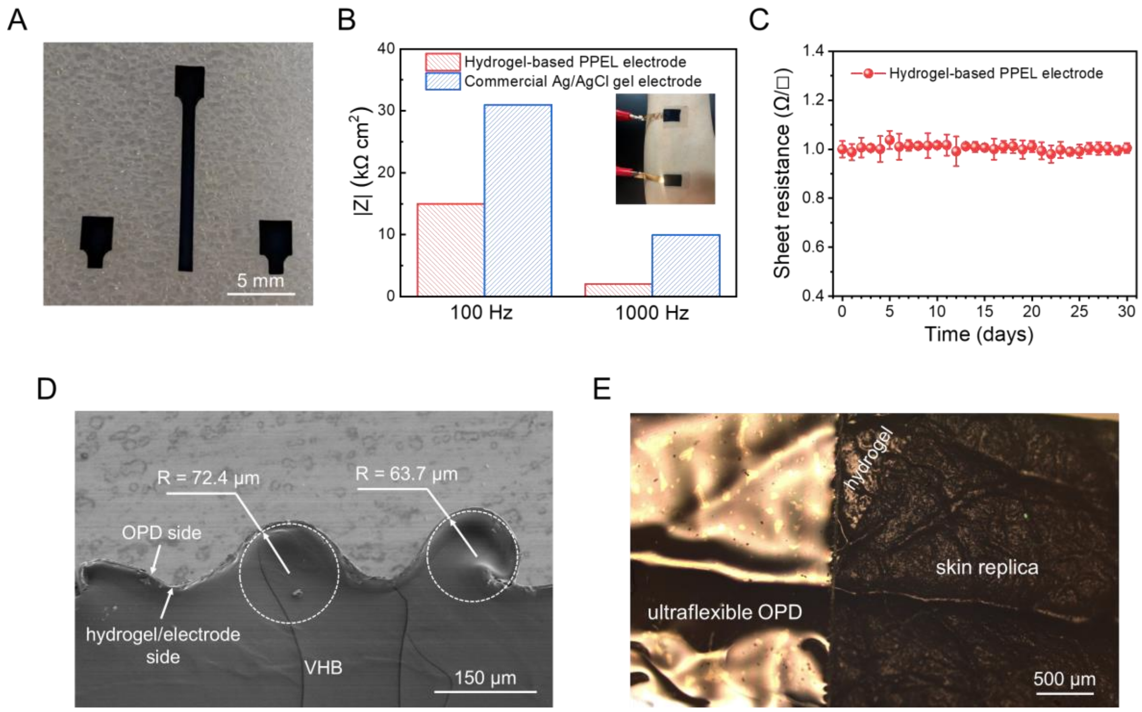

Figure S9. (A) Photograph showing the drop-cast PPEL electrodes on an ultrathin hydrogel substrate. The sample is placed on a white foam plate. (B) Skin impedance comparison between the drop-cast conductive polymer on hydrogel (red) and commercial Ag/AgCl gel electrode (blue). Inset shows the testing configuration using two shaped electrodes with an active area of 1 cm^2 for each and an inter-distance of 5.5 cm, adhered to the forearm of a male volunteer. (C) Sheet resistance of the conductive polymer on hydrogel recorded for 5 days. (D) Scanning electron microscopy image of the OPD/hydrogel/electrodes integrated patch attach to a

compressed VHB surface. (E) A microscopic image showing the high mechanical compliance of the integrated patch on the skin replica without forming interfacial air gaps.

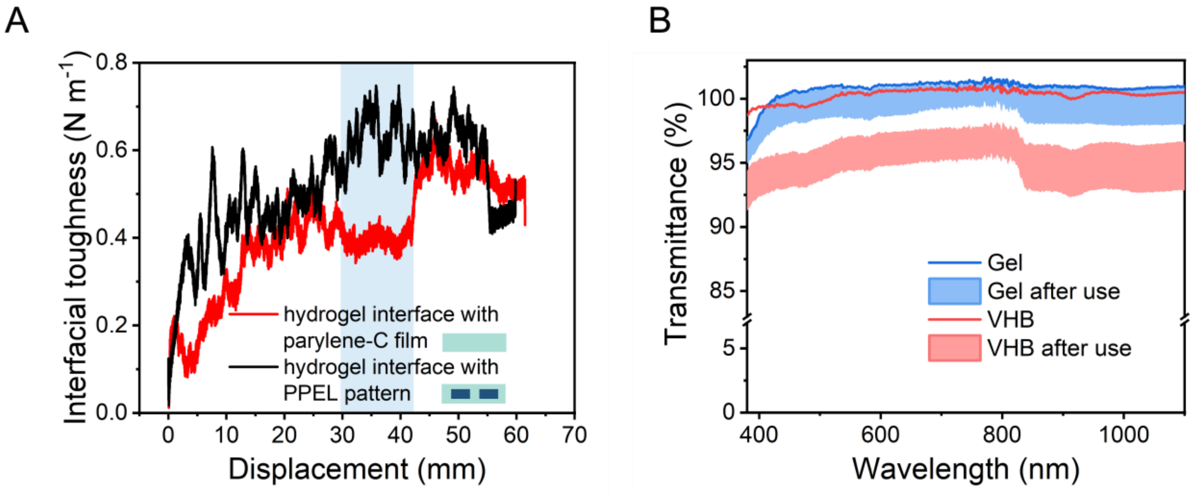

Figure S10. (A) Representative curves of the peeling force versus displacement. Comparison is made between a freshly prepared 10 μm thick hydrogel (red) and a 10 μm thick hydrogel after drop-casting conductive polymer pattern (black). (B) Transmittance of ultrathin hydrogel and 3M VHB 4905 tape before/after 5 times adhesion to palm skin, the light blue and light red area are the transmittance decrease result from 5 subjects after repeatedly adhesion test.

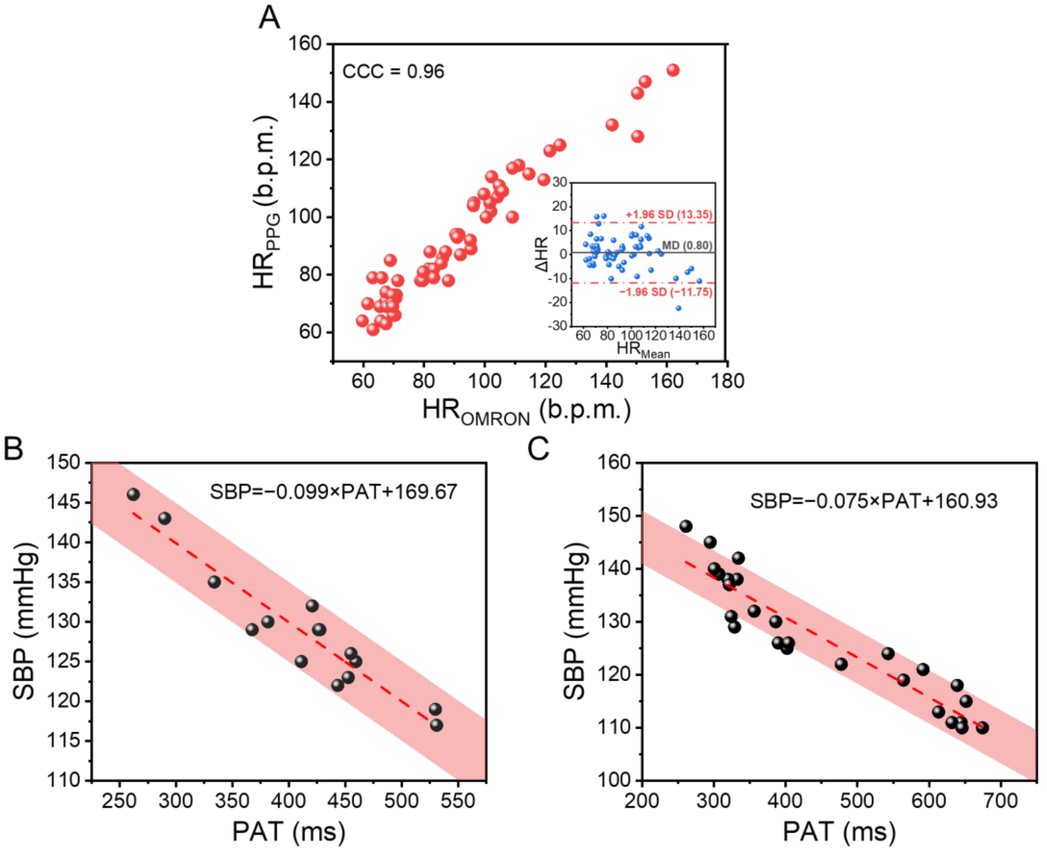

Figure S11. (A) Correlation plot of the heart rate measured by the skin-integrated PPG and a commercial sphygmomanometer (OMRON, J710). (Inset: Bland-Altman plot for heart rate collected from three healthy adults using PPG sensors and OMRON J710). (B-C) The correlation between PAT obtained by our PPG-ECG integrated system and SBP measured directly using an OMRON J710 sphygmomanometer. A linear fitting model was used to estimate blood pressure values. The margin of error (light red area) for the estimation model was set at ± 5 mmHg, which was deemed acceptable based on scientific guidelines for non-invasive blood pressure measurement. Data recorded from volunteer #13 and #1 are presented here in (B) and in (C), respectively, and from volunteer #21 are presented in main text **Figure 5H**. Detailed information for the volunteers are summarized in **Table S4**.

Table S1. Summary on the performance of recently reported NIR organic photodiodes.

Ref.	Substrate	Actice Layer	λ_{det} (nm)	V_{bias} (V)	R (A/W)	J_d (A/cm^2)	D_{sh}^* (Jones)
[6]	Glass	PTB7-Th:CO1-4Cl	900	−1	0.49*	<1 × 10^{-8}	5.82 ×10^{12} b)
[7]	Glass	PTB7-Th: COTIC-4Cl:$PC_{71}BM$	1050	−0.1	0.35	1.1 × 10^{-8}	5 × 10^{12} b)
[8]	Glass	PDT:$PC_{61}BM$	900	−1	0.037	1.96 × 10^{-9}	1.9 × 10^{12}
[9]	Glass	P1:$PC_{71}BM$	1150	−2	0.08	7 × 10^{-4} a)	2.2 × 10^{11}
[10]	Glass	PMDPP3T:$PC_{61}BM$	850	−0.2	0.35	2.98 × 10^{-9}	1.23 × 10^{13}
[11]	Glass	FDT:$PC_{61}BM$	900	0	> 0.3*	~5 × 10^{-10} a)	>2×10^{13}
[12]	Glass	CS-DP:$PC_{71}BM$	850	0	0.33	4.25 × 10^{-10}	5.73 × 10^{13}
[13]	Glass	PbPc/PbPc:C_{70} /C_{70}	890	0	0.22*	~2 × 10^{-8} a)	4.2 × 10^{12} b)
[14]	Glass	PDPPSDTPS: $PC_{60}BM$	1000	0	~ 0.40^a	~1 × 10^{-7} a)	N/A
[15]	Glass	P1:CA:$PC_{71}BM$	1100	0<br>−1	~ 0.23*<br>~ 0.31*	N/A<br>~6 × 10^{-5} a)	1.2 × 10^{11} b)<br>N/A
[16]	Glass	PDTPN-β:$PC_{61}BM$	900	−0.1	0.18	6.1 × 10^{-8}	1.29 × 10^{12}
[17]	Glass	PTB7-Th:CO1-4Cl	920	0<br>−2	0.50<br>0.53	2 × 10^{-10}<br>2 × 10^{-7}	>2 × 10^{13}<br>>2 × 10^{12}
[18]	Glass	PTB7-Th:COTIC-4F	970<br>920	0<br>0	0.37<br>0.46	1.4 × 10^{-8}<br>8.9 × 10^{-10}	1.7 × 10^{11}<br>1.5 × 10^{12}
[19]	Glass	PTTBAI:$P_{71}BM$	1100	−2	~ 0.27*	2 × 10^{-7}	>10^{12}

[20]	Glass	PTB7-Th:COTIC-4F	1050<br>850	−2<br>−2	0.25<br>0.4	5×10^{-9}<br>2×10^{-10}	6×10^{12}<br>5.6×10^{13}
[21]	Glass	PbPc:C_{60}	970	−3	0.24	9.97×10^{-6}	1.36×10^{11}
[22]	Glass	PTB7-Th: CO_i8DFIC:$PC_{71}BM$	900	0	0.37	N/A	5.6×10^{11} [b)]
[23]	PDMS	PM6:Y6	850	0	0.54	5.9×10^{-10}	1.67×10^{13} [b)]
[24]	PET	SQ-H:$PC_{61}BM$	1050	0	0.07	6×10^{-9} [a)]	4×10^{10} [b)]
This work	Parylene	PTB7-Th: COTIC-4F: $PC_{71}BM$	1020	0<br>−1	0.53<br>0.58	3.8×10^{-10}<br>1.3×10^{-7}	4.8×10^{13}<br>2.9×10^{12}

[a)] Data observed from figures. [b)] D^* measured based actual noise current.

Full name of the abbreviations inside the table are listed below:

PTB7-Th: poly[4,8-bis(5-(2-ethylhexyl)thiophen-2-yl)benzo[1,2-b;4,5-b′]dithiophene-2,6-diyl-alt-(4-(2-ethylhexyl)-3-fluorothieno[3,4-b]thiophene-)-2-carboxylate-2,6-diyl)]

CO1-4Cl: 2-((Z)-2-((5-(6-(5-((Z)-(1-(dicyanomethylene)-5,6-dichloro-3-oxo-1H-inden-2(3H)-ylidene)methyl)-3-((2-ethylhexyl)oxy)thiophen-2-yl)-4,4-bis(2-ethylhexyl)-4H-cyclopenta[1,2-b:5,4-b']dithiophen-2-yl)-4-(2-ethylhexyl)thiophen-2-yl)methylene)-5,6-dichloro-3-oxo-2,3-dihydro-1H-inden-1-ylidene)malononitrile

COTIC-4Cl: 2,2'-((2Z,2'Z)-((5,5'-(4,4-bis(2-ethylhexyl)-4H-cyclopenta[1,2-b:5,4-b']dithiophene-2,6-diyl)bis(4-((2-ethylhexyl)oxy)thiophene-5,2-diyl))bis(methanylylidene))bis(5,6-dichloro-3-oxo-2,3-dihydro-1H-indene-2,1-diylidene))dimalononitrile

$PC_{71}BM$: [6,6]-phenyl C71-butyric acid methyl ester

PDT: a D-A conjugated polymers consisting of dithienobenzotrithiophene and thienoisoindigo

$PC_{61}BM$: [6,6]-phenyl C61-butyric acid methyl ester

P1: polymer 1, P1 in ref (10) and (16) does not refer to the same polymer, details can be referred to the literature.

PMDPP3T: poly[[2,5-bis(2-hexyldecyl-2,3,5,6-tetrahydro-3,6-dioxopyrrolo[3,4-c]pyrrole-1,4-diyl]-alt-[3′,3″-dimethyl-2,2′:5′,2″-terthiophene]-5,5″-diyl]

FDT: 6,6-bis(2-ethylhexyl)-1,1′,3,3′-tetraphenyl-4,6-dihydro-1H-fluoreno[2,3,8,9]-1,2,4-ditriazin-4-yl

CS-DP: a A–π_2–D–π_1–D–π_2–A dimeric porphyrin small molecule

PbPc: lead phthalocyanine

PDPPSDTPS: a polymer composed of strong electron-accepting diketopyrrolopyrrole (DPP) moieties alternating with very strong electron-donating pyrrole-based groups

CA: camphoric anhydride, 1,8,8-trimethyl-3-oxabicyclo[3.2.1]octane-2,4-dione

PDTPN-β: 3-(5-(6-methyl-4-(naphthalen-2-yl)-4H-dithieno[3,2-b:2',3'-d]pyrrol-2-yl)thiophen-2-yl)-6-(5-methylthiophen-2-yl)-2,5-bis(2-octyldodecyl)-2,5-dihydropyrrolo[3,4-c]pyrrole-1,4-dione

COTIC-4F: 2,2'-((2Z,2'Z)-((5,5'-(4,4-bis(2-ethylhexyl)-4H-cyclopenta[1,2-b:5,4-b']dithiophene-2,6-diyl)bis(4-((2-ethylhexyl)oxy)thiophene-5,2-diyl))bis(methanylylidene))bis(5,6-difluoro-3-oxo-2,3-dihydro-1H-indene-2,1-diylidene))dimalononitrile

PTTBAI: alternating copolymer (P) of TT: thieno[3,2-b]thiophene and BAI: 7,14-bis(4-(2-octyldodecyl)thiophen-2-yl)diindolo[3,2,1-de:3',2',1'-ij][1,5]naphthyridine-6,13-dione

CO$_i$8DFIC: 2,2'-[[4,4,11,11-tetrakis(4-hexylphenyl)-4,11-dihydrothieno[2',3':4,5]thieno[2,3-d]thieno[2'''',3'''':4''',5''']thieno[2''',3''':4'',5'']pyrano[2'',3'':4',5']thieno[2',3':4,5]thieno[3,2-b]pyran-2,9-diyl]bis[methylidyne(5,6-difluoro)]]

PM6: poly[(2,6-(4,8-bis(5-(2-ethylhexyl-3-fluoro)thiophen-2-yl)-benzo[1,2-b:4,5-b′]dithiophene))-alt-(5,5-(1′,3′-di-2-thienyl-5′,7′-bis(2-ethylhexyl)benzo[1′,2′-c:4′,5′-c′]dithiophene-4,8-dione)]

Y6: 2,2′-((2Z,2′Z)-((12,13-bis(2-ethylhexyl)-3,9-diundecyl12,13-dihydro-[1,2,5]thiadiazolo[3,4-e]thieno[2″,3″:4′,5′]thieno[2′,3′:4,5]pyrrolo[3,2-g]thieno[2′,3′:4,5]thieno[3,2-b]indole-2,10-diyl)bis(methanylylidene))bis(5,6-difluoro-3-oxo2,3-dihydro-1H-indene-2,1-diylidene))dimalononitrile

SQ-H: (Z)-3-(dicyanomethylene)-4-((1-dodecylquinolin-1-ium-4-yl)methylene)-2-(((E)-1-dodecylquinolin-4(1H)-ylidene)methyl)cyclobut-1-en-1-olate

Table S2. GIWAXS parameters of COTIC-4F, PTB7-Th, PTB7-Th:COTIC-4F and PTB7-Th:COTIC-4F:PC$_{71}$BM based films in OOP direction.

Sample	OOP (100) q (Å^{-1})	Distance (Å)	FWHM (Å^{-1})	Intensity	CCL (Å)
COTIC-4F (in CB)	0.42 ± 0.01	14.96	0.06 ± 0.01	255.9	94.25

COTIC-4F (in CB+CN)	0.42 ± 0.01	14.96	0.13 ± 0.01	30.7	43.50
PTB7-Th	0.34 ± 0.01	18.48	0.22 ± 0.01	153.5	25.70
Binary	0.32 ± 0.01	19.64	0.13 ± 0.01	274.8	43.50
Ternary	0.32 ± 0.01	19.64	0.13 ± 0.01	282.7	43.50
**Sample**	**OOP (010) q (Å^{-1})**	**Distance (Å)**	**FWHM (Å^{-1})**	**Intensity**	**CCL (Å)**
COTIC-4F (in CB)	1.80 ± 0.01	3.49	0.13 ± 0.01	5.5	43.50
COTIC-4F (in CB+CN)	1.82 ± 0.01	3.45	0.15 ± 0.01	18.9	37.70
PTB7-Th	1.55 ± 0.01	4.05	0.39 ± 0.01	175.3	14.50
Binary	1.77 ± 0.01	3.55	0.22 ± 0.01	183.0	25.70
Ternary	1.76 ± 0.01	3.57	0.20 ± 0.01	156.5	28.27

Table S3. GIWAXS parameters of COTIC-4F, PTB7-Th, PTB7-Th:COTIC-4F and PTB7-Th:COTIC-4F:PC$_{71}$BM based films in IP direction.

**Sample**	**IP (100) q (Å^{-1})**	**Distance (Å)**	**FWHM (Å^{-1})**	**Intensity**	**CCL (Å)**
COTIC-4F (in CB)	0.35 ± 0.01	17.95	0.03 ± 0.01	163.1	188.50
COTIC-4F (in CB+CN)	0.36 ± 0.01	17.45	0.04 ± 0.01	52.3	141.37
PTB7-Th	0.27 ± 0.01	23.27	0.09 ± 0.01	516.0	62.83
Binary	0.31 ± 0.01	20.27	0.11 ± 0.01	412.0	51.41
Ternary	0.31 ± 0.01	20.27	0.11 ± 0.01	422.0	51.41
**Sample**	**IP(010) q (Å^{-1})**	**Distance (Å)**	**FWHM (Å^{-1})**	**Intensity**	**CCL (Å)**
COTIC-4F (in CB)	1.80 ± 0.01	3.49	0.13 ± 0.01	14.0	43.50

COTIC-4F (in CB+CN)	1.77 ± 0.01	3.55	0.10 ± 0.01	3.6	56.55

Table S4. Characteristics of the 26 volunteers who participated in this study.

SUBJECT	GENDER	AGE	REGION	SKIN TONE
1*	Male	25	East Asian	
2	Female	21	East Asian	
3	Female	23	East Asian	
4	Male	26	East Asian	
5	Male	24	East Asian	
6	Female	25	East Asian	
7	Male	24	East Asian	
8	Female	23	East Asian	
9	Male	24	East Asian	
10	Male	26	East Asian	
11	Female	22	East Asian	
12	Male	23	East Asian	
13*	Male	27	East Asian	
14	Female	55	East Asian	
15	Female	56	East Asian	
16	Male	63	East Asian	
17	Female	24	Southeast Asian	
18	Female	23	Southeast Asian	
19	Female	25	South Asian	
20	Female	24	East Asian	
21*	Male	31	East Asian	
22	Male	26	East European	
23	Male	28	Central African	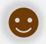
24	Male	26	East European	
25	Male	27	East European	
26	Male	31	Central African	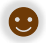

*: volunteers who additionally participated in the blood-pressure measurement experiments.

SI References

[1] a) Y. Khan, D. Han, A. Pierre, J. Ting, X. Wang, C. M. Lochner, G. Bovo, N. Yaacobi-Gross, C. Newsome, R. Wilson, A. C. Arias, *Proc. Natl. Acad. Sci. U.S.A.* **2018**, 115, E11015; b) M. Nitzan, A. Romem, R. Koppel, *Med. Devices (Auckl.)* **2014**, 7, 231.
[2] E. O. Polat, G. Mercier, I. Nikitskiy, E. Puma, T. Galan, S. Gupta, M. Montagut, J. J. Piqueras, M. Bouwens, T. Durduran, G. Konstantatos, S. Goossens, F. Koppens, *Sci. Adv.* **2019**, 5, eaaw7846.
[3] S. Chatterjee, P. A. Kyriacou, presented at *2019 41st Annual International Conference of the IEEE Engineering in Medicine and Biology Society (EMBC)*, 23-27 July 2019, **2019**.
[4] J. G. Webster, *Design of pulse oximeters*, CRC Press, **1997**.
[5] C. Orphanidou, in *Signal Quality Assessment in Physiological Monitoring: State of the Art and Practical Considerations*, Springer International Publishing, Cham 2018.
[6] J. Huang, J. Lee, H. Nakayama, M. Schrock, D. X. Cao, K. Cho, G. C. Bazan, T.-Q. Nguyen, *ACS Nano* **2021**, 15, 1753.
[7] Z. Zhong, F. Peng, L. Ying, G. Yu, F. Huang, Y. Cao, *Sci. China Mater.* **2021**, 64, 2430.
[8] J. Han, J. Qi, X. Zheng, Y. Wang, L. Hu, C. Guo, Y. Wang, Y. Li, D. Ma, W. Qiao, Z. Y. Wang, *J. Mater. Chem. C* **2017**, 5, 159.
[9] J. Han, D. Yang, D. Ma, W. Qiao, Z. Y. Wang, *Adv. Opt. Mater.* **2018**, 6, 1800038.
[10] S. Xiong, J. Tong, L. Mao, Z. Li, F. Qin, F. Jiang, W. Meng, T. Liu, W. Li, Y. Zhou, *J. Mater. Chem. C* **2016**, 4, 1414.
[11] Y. Zheng, M.-s. Miao, G. Dantelle, N. D. Eisenmenger, G. Wu, I. Yavuz, M. L. Chabinyc, K. N. Houk, F. Wudl, *Adv. Mater.* **2015**, 27, 1718.
[12] L. Xiao, S. Chen, X. Chen, X. Peng, Y. Cao, X. Zhu, *J. Mater. Chem. C* **2018**, 6, 3341.
[13] Z. Su, F. Hou, X. Wang, Y. Gao, F. Jin, G. Zhang, Y. Li, L. Zhang, B. Chu, W. Li, *ACS Appl. Mater. Interfaces* **2015**, 7, 2529.
[14] K. H. Hendriks, W. Li, M. M. Wienk, R. A. J. Janssen, *J. Am. Chem. Soc.* **2014**, 136, 12130.
[15] Z. Wu, Y. Zhai, W. Yao, N. Eedugurala, S. Zhang, L. Huang, X. Gu, J. D. Azoulay, T. N. Ng, *Adv. Funct. Mater.* **2018**, 28, 1805738.
[16] Z. Liu, Y. Chen, Y. Hu, J. Dong, J. Wen, J. Gao, P. Li, *Polymer* **2021**, 223, 123728.
[17] J. Huang, J. Lee, J. Vollbrecht, V. V. Brus, A. L. Dixon, D. X. Cao, Z. Zhu, Z. Du, H. Wang, K. Cho, G. C. Bazan, T.-Q. Nguyen, *Adv. Mater.* **2020**, 32, 1906027.
[18] J. Lee, S.-J. Ko, H. Lee, J. Huang, Z. Zhu, M. Seifrid, J. Vollbrecht, V. V. Brus, A. Karki, B. R. Luginbuhl, H. Wang, K. Cho, T.-Q. Nguyen, G. C. Bazan, *ACS Energy Lett.* **2019**, 4, 1732.
[19] F. Verstraeten, S. Gielen, P. Verstappen, J. Kesters, E. Georgitzikis, J. Raymakers, D. Cheyns, P. Malinowski, M. Daenen, L. Lutsen, K. Vandewal, W. Maes, *J. Mater. Chem. C* **2018**, 6, 11645.
[20] W. Yang, W. Qiu, E. Georgitzikis, E. Simoen, J. Serron, J. Lee, I. Lieberman, D. Cheyns, P. Malinowski, J. Genoe, H. Chen, P. Heremans, *ACS Appl. Mater. Interfaces* **2021**, 13, 16766.
[21] M.-S. Choi, S. Chae, H. J. Kim, J.-J. Kim, *ACS Appl. Mater. Interfaces* **2018**, 10, 25614.
[22] W. Li, Y. Xu, X. Meng, Z. Xiao, R. Li, L. Jiang, L. Cui, M. Zheng, C. Liu, L. Ding, Q. Lin, *Adv. Funct. Mater.* **2019**, 29, 1808948.
[23] N. Cui, Y. Song, C.-H. Tan, K. Zhang, X. Yang, S. Dong, B. Xie, F. Huang, *npj Flex. Electron.* **2021**, 5, 31.

[24] J. H. Kim, A. Liess, M. Stolte, A.-M. Krause, V. Stepanenko, C. Zhong, D. Bialas, F. Spano, F. Würthner, *Adv. Mater.* **2021**, 33, 2100582.
